# Mechanisms Underlying the Effects of Lianhua Qingwen on Sepsis-Induced Acute Lung Injury: A Network Pharmacology Approach

**DOI:** 10.3389/fphar.2021.717652

**Published:** 2021-10-14

**Authors:** Ruhao Yang, Haizhen Yang, Jie Wei, Wenqiang Li, Fang Yue, Yan Song, Xin He, Ke Hu

**Affiliations:** ^1^ Department of Respiratory and Critical Care Medicine, Renmin Hospital of Wuhan University, Wuhan, China; ^2^ Department of Emergency, Renmin Hospital of Wuhan University, Wuhan, China

**Keywords:** Lianhua Qingwen, acute lung injury, p53, apoptosis, network pharmacology

## Abstract

**Background and Purpose:** Sepsis is a life-threatening condition associated with secondary multiple organ injury. Acute lung injury (ALI) caused by sepsis has high morbidity and mortality in critical care units. Lianhua Qingwen (LHQW) is a traditional Chinese medicine composing of 11 herbs and 2 medicinal minerals. LHQW exhibits anti-inflammatory activity and is effective in treating pneumonia. Our study aimed to evaluate the effect of LHQW on sepsis-induced ALI and its underlying mechanism.

**Materials and Methods:** A network pharmacology approach was used to predict the bioactive components and effective targets of LHQW in treating ALI. We established ALI model C57/BL6 mice *via* an intraperitoneal injection of LPS and inhibited p53 expression by pifithrin-*α*, in order to validate the mechanism by which LHQW exerted protective role in ALI. Hematoxylin-eosin staining was conducted to assess the severity of lung injury. The severity of inflammation was evaluated based on MPO (myeloperoxidase) activity. TUNEL assay was employed to detect apoptotic cells. The levels of p53 and caspase-3 were tested by immunohistochemical staining and Western blotting. The expression levels of Bcl-2, Bax, cytochrome C and caspase-9 were detected by Western blotting.

**Results:** A total of 80 genes were associated with LHQW in the treatment of ALI. After PPI network construction, four active components (quercetin, luteolin, kaempferol and wogonin) and 10 target genes (AKT1, TP53, IL6, VEGFA, TNF, JUN, STAT3, MAPK8, MAPK1, and EGF) were found to be essential for ALI treatment. GO and KEGG analyses indicated that apoptosis pathway was mainly involved in the LHQW-ALI network. Animal experiments showed that LHQW was able to attenuate LPS-induced ALI, and medium-dose LHQW exhibited the most prominent effect. LHQW could inhibit the overexpression of p53 induced by LPS and suppress p53-mediated intrinsic apoptotic pathways by decreasing the levels of Bax, caspase-3 and caspase-9, increasing the expression of Bcl-2, and attenuating the release of cytochrome C in ALI mice.

**Conclusion:** This study reveals that LHQW may alleviate LPS-induced ALI *via* inhibiting p53-mediated intrinsic apoptosis pathways.

## Introduction

Acute lung injury (ALI) is relatively severe lung damage with edema, hyperemia, and inflammatory cell infiltration. The annual mortality rate of ALI is 4% among all hospital admissions, which accounts for 10% of all intensive care unit (ICU) admissions (Bellani et al., 2016). At present, there is no any specific effective treatment for ALI. Although the etiology and pathogeneis of ALI are complex, non-pulmonary sepsis is recognized as the most common predisposing factor for this disease, with an incidence rate of nearly 31% (Bersten et al., 2002). In this study, an animal model of sepsis-induced ALI was established by directly injecting lipopolysaccharide (LPS) into mice (Chen et al., 2010).

Lianhua Qingwen (LHQW) is a novel Chinese patent medicine, which has been commonly prescribed to treat influenza (Duan et al., 2011; Ding et al., 2017), acute upper and lower respiratory tract infection (Dong et al., 2014). As to COVID-19, LHQW also exhibits a good performance (Hu et al., 2021). LHQW derives from three classical prescriptions, such as “Maxing Shigan-Tang” in Han dynasty, “Da Huang” in Ming dynasty and “Yinqiao-San” in Qing dynasty. LHQW consists of 11 herbs and two medicinal minerals, including *Forsythia suspensa (Thunb.) Vahl* (Lianqiao, LQ), *Lonicera japonica Thunb.* (Jinyinhua, JYH), *Ephedra sinica Stapf* (Mahuang, MH), *Isatis tinctoria L.* (Banlangen, BLG), *Pogostemon cablin (Blanco) Benth.* (Guanghuoxiang, GHX), *Rheum palmatum L.* (Dahuang, DH), *Glycyrrhiza uralensis Fisch.* (Gancao, GC), *Dryopteris crassirhizoma Nakai* (Mianmaguanzhong, GZ), *Rhodiola crenulata (Hook.f. and Thomson) H. Ohba* (Hongjingtian, HJT), *Houttuynia cordata Thunb.* (Yuxingcao, YXC), *Prunus sibirica L.* (Kuxingren, KXR), *Gypsum* and *l-Menthol*. In the theory of traditional medicine (TCM), compound medicines are generally considered to exert synergistic effects on distinct targets. Besides, the complex etiologies also suggest that a combination of medicines may play better therapeutic activities if the dosage, proportion and compatibility of the compounds are optimized. Therefore, we believe that the therapeutic effects of TCM can benefit from the synergistic actions of compound medicines.

Network pharmacology is a novel approach to elucidate the complex pathophysiological progress by assessing the interactions among herbs, components, targets genes and diseases (Hopkins, 2007), which aids in understanding the instinct rules of complex formulas and revealing the multiple targets for TCM actions (Kibble et al., 2015). In this research, we utilized the network pharmacology approach to predict the effective targets between LHQW and ALI. We systematically demonstrated that apoptotic signal transduction in biological processes was crucial pathway for LHQW in the treatment of ALI through Gene Ontology (GO) enrichment. Moreover, TP53 was identified as one of 10 key genes according to protein-protein interaction (PPI) network analysis. These results indicated that LHQW might protect lung tissues from ALI by inhibiting p53-mediated apoptosis. In mammalian cells, P53 transcriptional activation causes cell growth arrest and induces cell apoptosis (Liu and Chen, 2006). The p53-mediated cell apoptosis has two major pathways: extrinsic pathway and intrinsic pathway (Ashkenazi and Dixit, 1998). The extrinsic pathway is mainly triggered by the engagement of death receptors. The intrinsic apoptosis pathways are dominated by Bax, puma and noxa, which can lead to the release of cytochrome C from mitochondria as well as the activation of caspase-3 and -9. Several studies have suggested that p53-mediated cell apoptosis may play an essential role in mediating the pathogenesis of ALI.

Based on the network pharmacology results, we observed that LHQW alleviated ALI through inhibition of p53-mediated cell apoptosis. In this study, LPS-induced ALI mouse model was built to evaluate the therapeutic effects of LHQW. Furthermore, pifithrin-*α* (a p53 specific inhibitor) was adopted to verify the mechanism of action of LHQW against p53-mediated cell apoptosis. Our results demonstrated that LHQW could protect the lung tissues from ALI, thus making it a promising strategy for alleviating the lung injuries caused by pathogenic factors.

## Materials and Methods

### Lianhua Qingwen Information

LHQW were produced by Shijiazhuang Yiling Pharmaceutical Co., Ltd., China (B2001019). LHQW consists of 11 herbs and two mineral Chinese medicines. The contents and proportions, method of extraction and fingerprint of Lianhuaqingwen (UPLC) were provided (Supplementary Material).

### Collection of Herb Formulation Ingredienst and Absorption, Distribution, Metabolism, and Excretion Testing

The TCM systems pharmacology database (TCMSP, https://tcmspw.com/tcmsp.php v2.3) (Ru et al., 2014) was used to retrieve all 13 TCM compounds in LHQW. TCMSP contains 499 herbs registered in the Chinese pharmacopoeia (2010) with 12,144 ingredients. ADME (absorption, distribution, metabolism, and excretion) screening was performed to determine bioactive components that meet the following two requirements: 1) oral bioavailability ≥30% and drug-likeness ≥0.18. The target gene of bioactive compounds were retrieved from prior literature and TCM system pharmacology database. UniPortKB (https://www.uniprot.org) was used to standardize target proteins and covert protein names into gene names under “*Homo sapiens*” conditions.

### Acute Lung Injury Targets

GeneCards (Stelzer et al., 2016) is an integrative database that contains thorough information on all human genes (both known and predicted). OMIM (Amberger and Hamosh, 2017) is a publicly available database that provides comprehensive information on human genes and genetic disorders as well as the association between genotype and phenotype. The keyword of “acute lung injury” was used in GeneCards (https://www.genecards.org/; v5.0; July 27th, 2020) and OMIM (https://www.omim.org; updated on September 22nd, 2020). We finally obtained 348 genes in OMIM and 376 genes in GeneCards with “Relevance score” >=25.

### Network Construction and Protein-Protein Interaction Analysis

Herb-component-target network was constructed using the Cytoscape v3.7.1 (Shannon et al., 2003). There were 80 common targets of the main active ingredients and acute lung injury. To build a PPI network, the shared targets were processed by STRING v11.0b (https://string-db.org/) with a limitation to “*Homo sapiens*” and confidence score ≥0.7. The PPI network of 80 overlapped targets based on STRING data was generated by Cytoscape v3.7.1, which contained the degree values of nodes. The herb-component-overlapped target network was constructed by Cytoscape v3.7.1 to determine the major ingredients of LHQW for treating ALI. Molecular Complex Detection (MCODE), a Cytoscape plugin, was used to pick out the major dense region in the network of 80 overlapped target gene. The following settings were used: Degree Cut-off = 2, Node Score Cut-off = 0.2, K-Core = 2, and Max Depth = 100. The seed genes were obtained in protein clusters with highest degree of MCODE Score.

### Gene Ontology Enrichment and KEGG Pathway Analyses

To identify the functional characteristics of the putative targets for LHQW in ALI. GO enrichment and KEGG pathway analyses were performed by Metascape (http://metascape.org/; v3.5; last updated date on 2020-09-16), which contained a series of bioinformatics tools for analyzing gene/protein functions and improving pre-clinical decisions (Zhou et al., 2019). The “*H. sapiens”* and *p*-value cut-off of <0.01 were adjusted to obtain the analysis results. Cytoscape v3.7.1 was used to construct the pathway-target network.

### Animals

C57/BL6 mice (male, 8–10 weeks old) were supplied by Charles River Labs (Beijing, China). The ethical approval for animal experiments was obtained from the Animal Ethics Committee of Wuhan University, Wuhan, China (2019K-K081). Mice were maintained under specific pathogen-free (SPF) condition at 23 ± 2°C over a 12-h day/night cycle, with free access to water and food. A week of adjustable feeding was conducted prior to LHQW administration.

All mice were categorized into seven groups as follows: 1) control group (*n* = 4); 2) LPS (Sigma, L2630; 10 mg) group (5 mg/kg, *n* = 4); 3) LPS with low-dose LHQW (700 mg/kg/day, *n* = 4); 4) LPS with medium-dose LHQW (1,400 mg/kg/day, *n* = 4); 5) LPS with high-dose LHQW (2,800 mg/kg/day; *n* = 4); 6) LPS with pifithrin-*α* (TargetMol, T2707; 5 mg) group (3 mg/kg/day, *n* = 4); and 7) LPS with pifithrin-*α* and medium-dose LHQW (*n* = 4). LHQW was administrated by gavage for six successive days. On the fifth day, pifithrin-*α* was given by intraperitoneal injection for two successive days. On the sixth day, the mice were subjected to an intraperitoneal injection of LPS and then sacrificed after 24 h. The left lung tissues were snap-frozen in liquid nitrogen and kept at −80°C for Western blotting; while the right lung tissues were fixed in 10% paraformaldehyde for histological, immunohistochemical and TUNEL staining.

### Histology

After fixing in 10% paraformaldehyde and embedding in paraffin, the lung sections (4 μm) were stained with hematoxylin and eosin (HE). The histopathological changes in lung tissues were examined using an optical microscope. Lung injury score (Matute-Bello et al., 2011) was used to measure the histological evidence of tissue injury.

### Immunohistochemistry

The lung sections in each sample (3 μm) were deparaffinized and rehydrated with turpentine, ethanol and water. The sections were incubated overnight at 4°C with p53 polyclonal antibody (1:250 dilution; Immunoway, YT3528), caspase-3 polyclonal antibody (1:250 dilution; Poteintech, 19677-1-AP) or MPO (myeloperoxidase) (1:1000 dilution; Abcam, ab208670). On the next day, the sections were exposed to the corresponding secondary antibody at 37°C for 1 h. The positive cells were counted in three fields of a lung section.

### TUNEL Staining

The lung sections in each sample (3 μm) were deparaffinized and rehydrated with ethanol at graded concentrations of 100, 95, and 75% (v/v) each for 5 min. After rinsing with distilled water, proteinase K (10 μg/ml, Roche) was added to the lung sections for 10 min, and then washed again for three times in Tris-buffered saline (TBS; each time for 5 min). Equilibrium solution was added to each lung section for 10 min. TUNEL assay kit (Roche, 11684795910) was employed to determine the apoptotic rate of lung tissue. DAPI (Roche, 216276) was used to stain the nucleus. TUNEL-positive cells were counted in three fields of a lung section.

### Western Blotting

After scrapping, the lung tissue was lysed in RIPA lysis buffer for 30 min to isolate total protein. The lysate was transferred into an EP tube and centrifuged for 5 min at 12,000 rpm/min. Total protein content was assessed by BCA assay kit (Thermo Fisher Scientific). The protein samples were separated on 12% sodium dodecyl sulfate Tris-HCl gels according to their molecular weight, and then immobilized onto PVDF membranes *via* electroblotting. After blocking with 5% skimmed milk in TBS containing 0.1% Tween (TBST) for 1 h, the membrane was exposed to the primary antibody against p53 (Immunoway, YT3528), Bcl-2 (Biorbyt, orb10173), Bax (Proteintech, 50599-2-Ig), cytochrome C (immunoway, YM3402), caspase-3 (Proteintech, 19677-1-AP), caspase-9 (Proteintech, 66169-1-AP), VDAC (Abcepta, AP6627A) or GAPDH (GOOD HERE, AB-P-R 001) at 4°C for 24 h. After rinsing in TBST for five times (each for 5 min), the membrane was exposed to the corresponding secondary antibody for 2 h at 37°C. Super ECL Plus (Applygen Technologies, P1050) was added onto the membranes to visualize the protein bands. The intensity of each band was measured by ImageJ software.

### Statistical Analyses

All experiments were repeated for three times. All values were shown as mean ± SD. One-way ANOVA followed by Tukey’s post-hoc test was performed for comparing the differences between groups. *p* < 0.05 was considered statistically significant. All statistical data were calculated using the GraphPad Prism 8 software.

## Results

### Herb Compound-Predicted Target Network

There were 223 active compounds in LHQW after eliminating the duplicates from TCMSP database. A total of 38 active compounds were identified from BLG, 19 from KXR, 16 from DH, 92 from GC, 7 from GZ, 11 from GHX, 23 from JYH, 23 from LQ, 23 from MH, 7 from YXC, and 3 form HJT (Supplementary Table S1). Among the active compounds, 188 compounds contained the information about the target genes. There were 21 common ingredients for at least two herbs, in which two ingredients (mairin, luteolin) were common for three herbs, two ingredients (sitosterol, stigmasterol) were common for four herbs, one ingredient (beta-sitosterol) was common for five herbs, one ingredient (quercetin) was common for six herbs, and one ingredient (kaempferol) was common for seven herbs (Supplementary Figure S1). Finally, we identified 254 predicted targets after eliminating the duplicates (Supplementary Table S2).

### Lianhua Qingwen Related Targets-Acute Lung Injury Related Target Network

**FIGURE 1:**
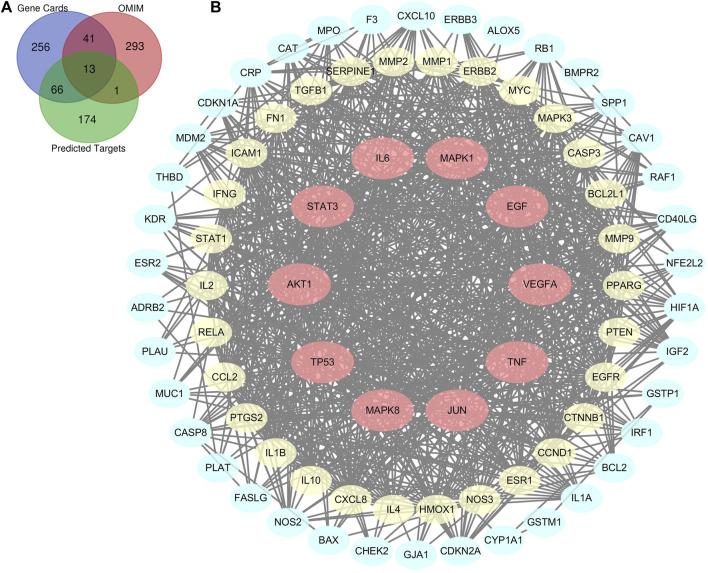
Bioinformatics analysis of overlapping genes. **(A)** 80 overlapping genes between the predicted targets of LHQW (254 genes) and the targets associated with ALI in GeneCards (376 genes) and OMIM databases (348 genes). **(B)** PPI network of the 80 overlapping genes. The importance of each gene was analyzed by STRING. The higher the degree was, the more crucial the gene was. Red nodes represent the major nodes with degree values ≥40. The colors of nodes from reddish to yellowish to blueish are arranged in descending order based on their degree values.

There were a total of 254 potential targets in LHQW and 670 ALI-related genes based on OMIM and GeneCards databases, and 80 overlapping genes were identified from the above information (Figure 1A). The 80 overlapping genes were imported into STRING to establish a compound-disease co-targets PPI network containing 80 nodes and 849 edges (Figure 1B). We identified that AKT1, TP53, IL6, VEGFA, TNF, JUN, STAT3, MAPK8, MAPK1, and EGF were pivotal genes with high degree values of ≥40 (Supplementary Table S3).

### Gene Ontology and KEGG Analyses of Overlapping Genes

**FIGURE 2:**
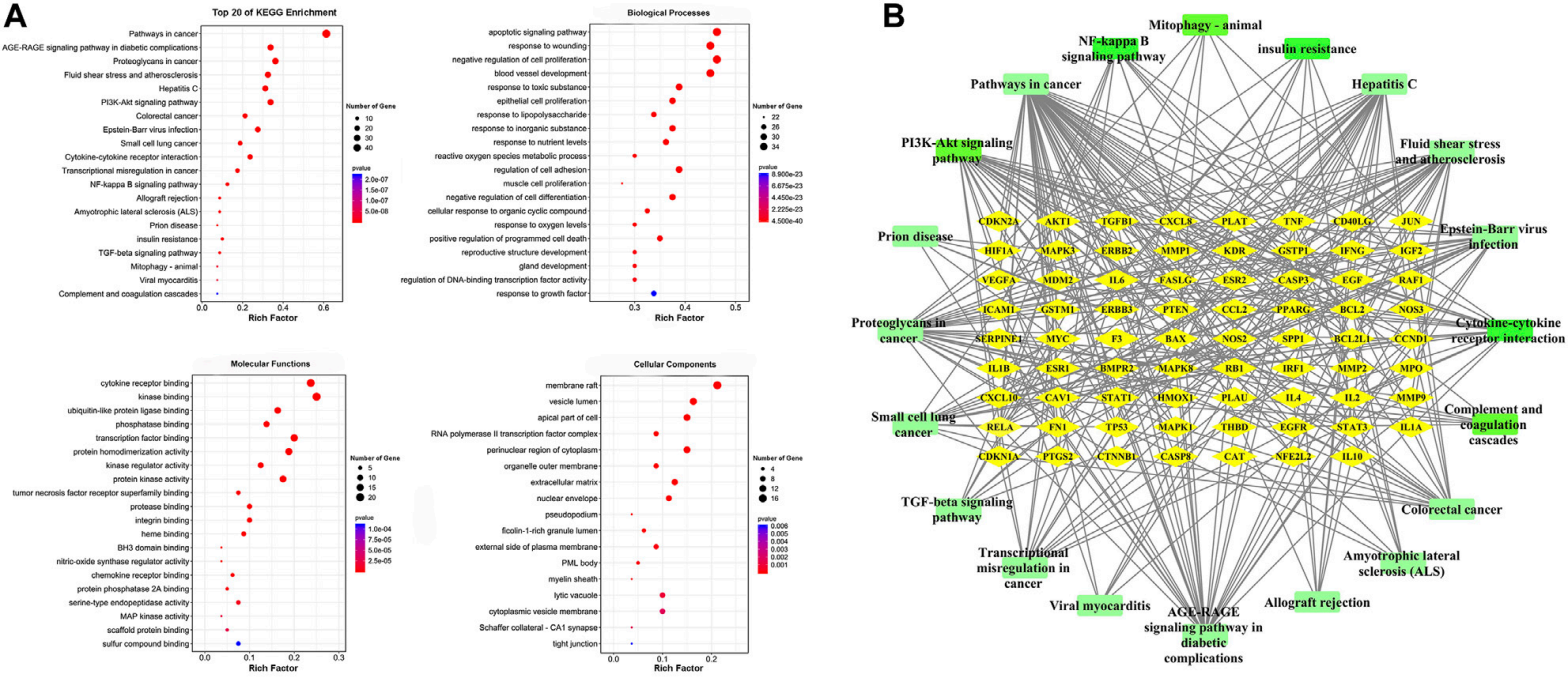
GO enrichment of the 80 common genes. **(A)** A bubble chart of top 20 GO enrichment terms. The redder the bubble, the smaller the *p*-value; the bigger the bubble, the greater the number of genes participated in this pathway. **(B)** PPI network of the top 20 KEGG signaling pathways and associated target genes. Green nodes represent top 20 KEGG pathways and yellow nodes indicate the genes participated. Among them, PI3K-Akt signaling pathway, NF-kappa B signaling pathway, Mitophagy-animal, Complement and coagulation cascades, Cytokine-cytokine receptor interaction, and TGF-beta signaling pathway were closely related to ALI occurrence.

GO analysis was carried out based on the above 80 matching genes of LHQW and ALI. The top 20 GO enrichment terms are shown (Figure 2A). For biological processes, the apoptotic signaling pathway (GO:0097190), response to wounding (GO:0009611), negative regulation of cell proliferation (GO:0008285), blood vessel development (GO:0001568), response to toxic substance (GO:0009636), epithelial cell proliferation (GO:0050673), response to lipopolysaccharide (GO:0032496), and others were enriched. For cellular components, the membrane raft (GO:0045121), vesicle lumen (GO:0031983), apical part of cell (GO:0045177), RNA polymerase II transcription factor complex (GO:0090575), perinuclear region of cytoplasm (GO:0048471) and others were enriched. For molecular functions, cytokine receptor binding (GO:0005126), kinase binding (GO:0019900), ubiquitin-like protein ligase binding (GO:0044389), phosphatase binding (GO:0019902), transcription factor binding (GO:0008134) and others were enriched. In addition, KEGG analysis was carried out to determine the crucial pathways associated with the 80 genes (Supplementary Table S4). The pathways, such as PI3K-Akt signaling pathway (hsa04151), complement and coagulation cascades (hsa04610), NF-κB signaling pathway (ko04065), cytokine-cytokine receptor interaction (ko04060), mitophagy-animal (ko04137) and TGF-*β* signaling pathway (ko04350), were related to ALI caused by LPS (Figure 2B).

### Cluster of Lianhua Qingwen Related Targets-ALI Related Target Network

**FIGURE 3:**
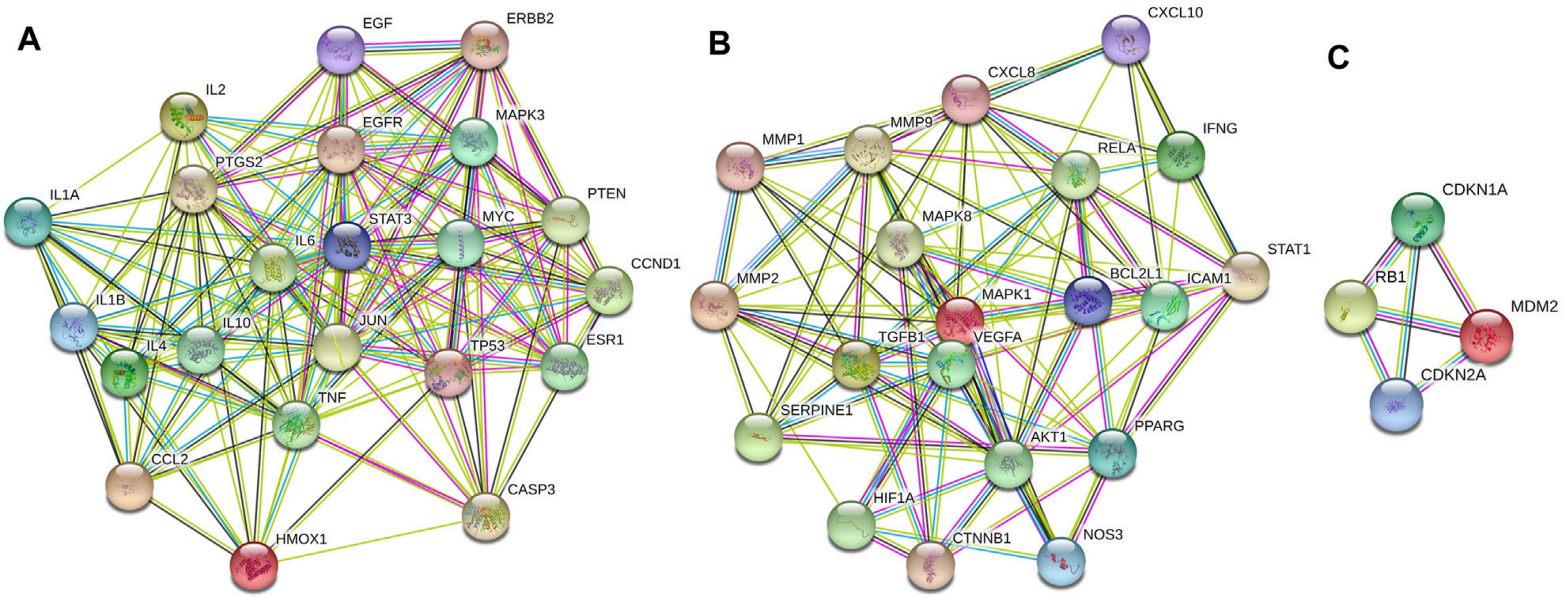
Cluster of the 80 overlapping genes-containing PPI network. Three clusters were identified. **(A–C)** stand for Clusters 1, 2, and 3, respectively. Cluster 1 had highest score of 15.238, and TP53 was identified as the seed gene. Cluster 2 had the score of 10.526, and MAPK8 was the seed gene. Cluster 3 had the score of 4.0, and MDM2 was the seed gene.

The information about 80 overlapping genes from STRING database were imported into Cytoscape for further analysis. MCODE was used to analyze the genes and generate 3 clusters (Figure 3). Cluster 1 had highest score of 15.238, which included TP53, CCL2, STAT3, MYC, ESR1, PTEN, MAPK3, EGFR, CASP3, CCND1, TNF, IL1B, EGF, IL6 and PTGS2. TP53 was identified as the seed gene. Cluster 2 had the score of 10.526. Cluster 2 included MAPK8, MMP1, CXCL8, BCL2L1, NOS3, ICAM1, IFNG, MMP2 and MMP9, and MAPK8 was the seed gene. Cluster 3 included MDM2,CDKN1A, RB1, CDKN2A, and had the score of 4.0. MDM2 was the seed gene (Supplementary Table S5).

### Component-Overlapping Target Genes Protein-Protein Interaction Network

To identify the most effective component in LHQW for ALI treatment, the component-overlapping target genes PPI network was established, and the degree value was calculated through network analysis. We found that quercetin was the major component in LHQW with the highest degree, followed by luteolin, kaempferol and wogonin, all with degree values >20 (Supplementary Figure S2).

### Lianhua Qingwen Prevents LPS-Induced ALI in Experimental Animals

**FIGURE 4:**
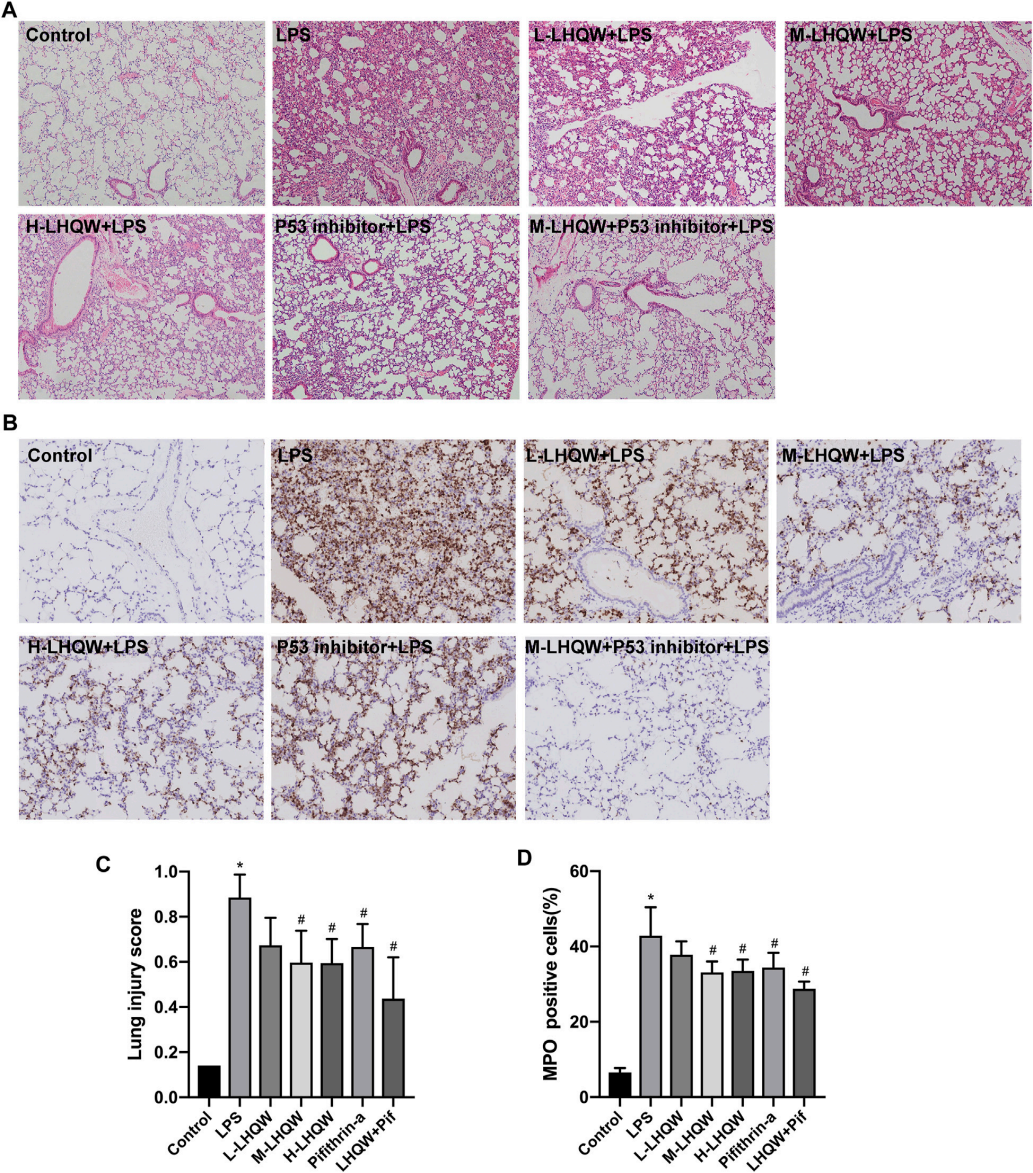
Effect of LHQW on histological features and MPO activity in ALI mice. **(A)** HE staining. Magnification, ×100 **(B)** The levels of MPO in mouse lung tissues from different groups. Brown staining indicates MPO-positive expression. Magnification, ×200 **(C)** Lung injury scores were measured in 20 random fields of lung sections according to HE staining (400 × total magnification), taken from three mice in each group. **(D)** The percentages of MPO-positive cells were counted in three fields of a lung section. LPS, 5 mg/kg; L-LHQW, 700 mg/kg/day; M-LHQW, 1,400 mg/kg/day; H-LHQW, 2,800 mg/kg/day; p53 inhibitor, pifithrin-*α* (3 mg/kg/day). Data are shown as mean ± SD from one-way ANOVA followed by Tukey’s post-hoc test. ^*^ *p* <0.05, versus Control; ^#^ *p* < 0.05, versus LPS.

To determine the effects of LHQW on LPS-induced ALI, we observed pathological and inflammatory changes in the lung section of ALI model mice. LHQW pretreatment was conducted before an intraperitoneal injection of LPS (5 mg/kg). Lung tissue were harvested after 24 h and subjected to HE staining and MPO-positive neutrophil assessment. There were significant pathological changes in the mice challenged with LPS. Thickened alveolar wall, increased neutrophils in the alveolar and interstitial space, and congestion of pulmonary capillary were observed in the LPS-challenged mice. Treatment with high- or medium-dose LHQW significantly alleviated the pathological changes and improved lung injury score in the LPS-challenged mice. However, there was no difference in pathological changes between the mice pretreated with high- and medium-dose LHQW (Figures 4A,C). The expression of MPO was also remarkably increased after LPS treatment, and then reduced after pretreatment with high- and medium-dose LHQW, which were consistent with the results of HE staining (Figures 4B,D).

### Lianhua Qingwen Alleviates ALI Damage by Suppressing p53 Overexpression in LPS-Induced ALI

**FIGURE 5:**
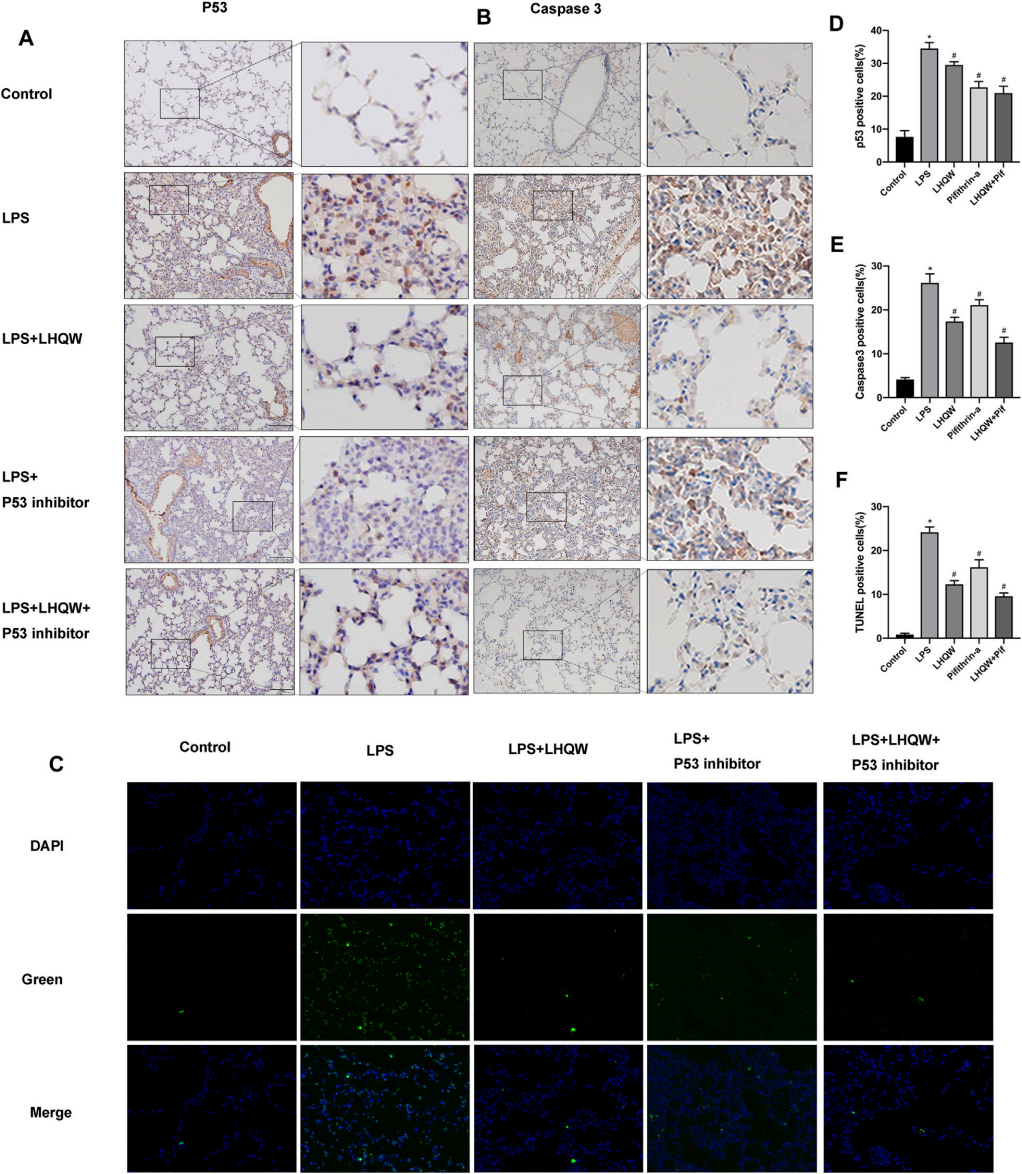
Effect of LHQW on apoptosis in ALI mice. **(A)** Representative images of immunohistochemistry for p53 in the mouse lung tissues from different groups. Brown staining indicates p53 expression. Magnification, ×200. **(B)** Representative images of immunohistochemistry for caspase-3 in the mouse lung tissues from different groups. Brown staining indicates caspase-3 expression. Magnification, ×200. **(C)** Lung tissue sections were subjected to TUNEL staining. TUNE-positive cells were green and indicated apoptotic cells. Magnification, ×400 **(D)** The percentages of p53-positive cells were counted in three fields of a lung section. **(E)** The percentages of caspase-3-positive cells were counted in three fields of a lung section. **(F)** The percentages of Tunel-positive cells were counted in three fields of a lung section. LPS, 5 mg/kg; LHQW, 1,400 mg/kg/day; p53 inhibitor, pifithrin-*α* (3 mg/kg/day). Data are shown as mean ± SD from one-way ANOVA followed by Tukey’s post-hoc test. ^*^ *p* < 0.05, versus Control; ^#^ *p* < 0.05, versus LPS.

To validate the key target gene p53 of LHQW predicted from the PPI networks, LPS-induced ALI mice were intraperitoneally injected with 3 mg/kg pifithrin-*α* (p53 inhibitor) for two successive days. We observed an increase in the expression of p53 in LPS-challenged mice by immunohistochemical staining, but the expression was decreased in mice treated with medium-dose LHQW or pifithrin-*α* (Figures 5A,D). Besides, LHQW and pifithrin-*α* also attenuated the pathological changes and MPO-positive neutrophils in the lung tissues of LPS-challenged mice. The decreased p53 expression and alleviated pathological changes were more obvious in mice treated with a combination of LHQW and pifithrin-*α* compared with those treated with LHQW or pifithrin-*α* alone. Although pifithrin-*α* could attenuate pathological injuries and inflammation in the lung tissues of LPS-challenged mice, the effects were less obvious in mice treated with medium- or high-dose LHQW (Figures 4A,B).

### Lianhua Qingwen Inhibits Apoptosis in LPS-Induced ALI

To elucidate the protective mechanism of LHQW in LPS-induced ALI, we focused on apoptosis signaling pathway that interacts with many other signaling pathways to regulate biological processes. The immunohistochemistry results demonstrated that LPS remarkably upregulated the expression of caspase-3, and medium-dose LHQW could suppress the increased expression of caspase-3 (Figures 5B,E). Moreover, TUNEL staining results showed that apoptotic cells were increased after LPS challenge, and medium-dose LHQW significantly attenuated LPS-induced apotosis (Figures 5C,F). We also found that pifithrin-*α* could decrease caspase-3 expression and apoptotic cells. The mice treated with a combination of medium-dose LHQW and pifithrin-*α* exhibited the lowest levels of caspase-3 expression and apoptotic cells.

### Lianhua Qingwen Suppresses p53-Mediated Intrinsic Apoptosis Pathways in LPS-Induced ALI

**FIGURE 6:**
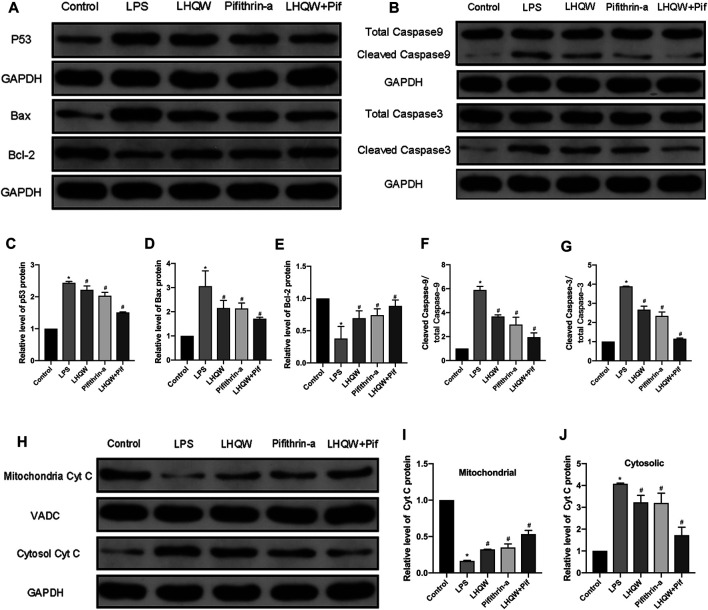
Effect of LHQW on p53-mediated intrinsic apoptosis. **(A)** Representative Western blot images of p53, Bax and Bcl-2. **(B)** Representative Western blot images of caspase-3 and caspase-9. **(C–G)** Comparison of the gray values of all proteins in mice by ImageJ software (with GAPDH loading control). **(H)** Representative Western blot images of cytochrome C in mitochondria and cytosol. **(I,J)** Comparison of the gray values of cytochrome C in mitochondria and cytosol in mice by ImageJ software (with GAPDH and VADC loading controls). LPS, 5 mg/kg; LHQW, 1,400 mg/kg/day; p53 inhibitor, pifithrin-*α* (3 mg/kg/day). Data are shown as mean ± SD from one-way ANOVA followed by Tukey’s post-hoc test. ^*^ *p* < 0.05, versus Control; ^#^ *p* < 0.05, versus LPS.

Given that LHQW could alleviate ALI damage by downregulating p53 expression and inhibiting apoptosis, the relationship between p53 and apoptosis was further investigated. In addition, p53 has been shown to mediate intrinsic apoptosis pathways. Thus, we assessed whether LHQW can attenuate LPS-induced ALI by inhibiting intrinsic apoptosis pathways caused by p53. The activities of intrinsic apoptosis-related proteins (p53, Bcl-2, Bax, cytochrome C, caspase-3 and caspase-9) were determined by Western blotting. The data revealed that LPS promoted the expression of p53, Bax, cytochrome C, caspase-3 and caspase-9, while suppressed the expression of Bcl-2 (Figures 6A–G) in the cytoplasm and inhibited the expression of cytochrome C in the mitochondria (Figures 6H–J). Interestingly, these effects were blunted by medium-dose LHQW and pifithrin-*α*, and it was more obvious in mice treated with combination therapy. Our findings imply that LHQW exhibits a protective effect on ALI mice by inhibiting p53-mediated intrinsic apoptosis pathways.

## Discussion

**FIGURE 7:**
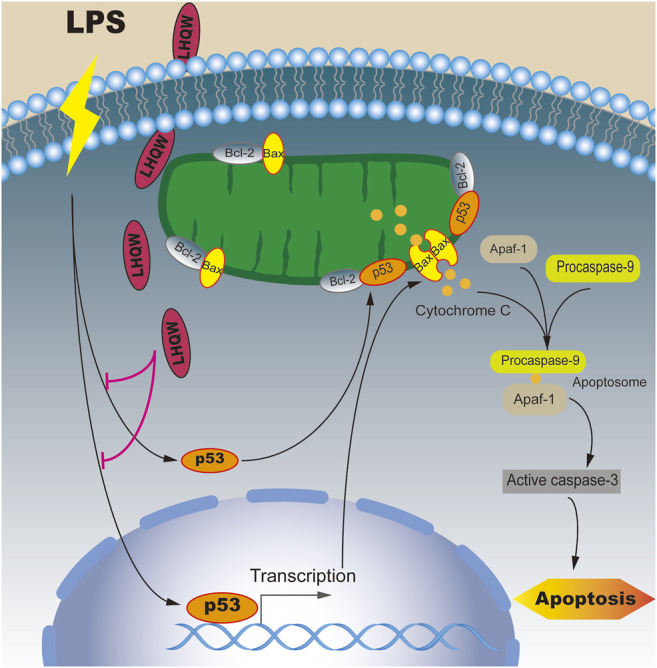
Mechanisms underlying the inhibitory effect of LHQW on p53-mediated apoptosis in ALI induced by LPS. Under normal conditions, Bcl-2 constant interacted with Bax and inhibited Bax from accumulating at the mitochondrial outer membrane, thus suppressing cell apoptosis. Under LPS stimulation, p53 and its downstream target Bax were upregulated and translocated to mitochondrial outer membrane to form a homodimer. On the other hand, p53 was able to permeabilize the outer mitochondrial membrane by binding to Bcl-2. The accumulation of Bax on the outer mitochondrial membrane could promote the release of cytochrome C, which in turn led to the formation of apoptosomes (Apaf-1, cytochrome C and caspase-9). Once activated, the effector caspases could induce cell apoptosis. LHQW treatment could attenuate LPS-induced apoptosis by downregulating p53 expression.

In this study, we found that medium- and high-dose LHQW could alleviate LPS-induced ALI *in vivo*. To elucidate the mechanisms of action, a network pharmacology-based approach was carried out. We found that apoptosis was the most important signaling pathway in biological processes and TP53 was one of 10 key genes for mediating the therapeutic effect of LHQW on ALI. More importantly, the apoptosis pathway is mainly regulated by p53. Therefore, we focused on the importance of p53-mediated apoptosis in the treatment progress of LPS-induced ALI. The results indicated that LHQW could ameliorate LPS-induced ALI by suppressing p53-mediated intrinsic apoptosis pathways *in vivo* (Figure 7).

ALI caused by sepsis is accounted for at least 40% of all cases (Wang et al., 2019). The lung is not only the earliest organ that gets affected but also contributes to the highest incidence for organ failure induced by sepsis. However, there is no effective treatment to stop the rapid progression of sepsis-induced ALI. Accumulating evidence has indicated that LHQW exerts therapeutic effects on acute respiratory infections caused by viruses. Compared with oseltamivir, LHQW could shorten the duration of symptoms caused by influenza, including fever, cough, sore throat, and body ache (Zhao et al., 2014). In an animal study, LHQW could also attenuate lung injury and viral load (Gao et al., 2020). LHQW also demonstrated satisfactory effects during the SARS outbreak in 2003 (Tong et al., 2004). Since December 2019, COVID-19 has emerged as a pandemic outbreak with greatly pathogenicity and transmissibility (Zhu et al., 2020), and several studies have shown that LHQW could reduce the course of COVID-19 and relieve the symptoms (Xiao et al., 2020). In Vero E6 cells, LHQW effectively blocked the replication of SARS-CoV-2 and decreased the production of pro-inflammatory cytokines (Runfeng et al., 2020). However, it remains elusive whether LHQW also exerts a protective effect on sepsis-induced pneumonia. In this study, the sepsis-induced ALI mouse model was constructed by an intraperitoneal injection of LPS. We found that medium- and high-dose LHQW could attenuate LPS-induced ALI in mice.

To further elucidate the mechanism by which LHQW prevented LPS-induced ALI, we analyzed active component and target genes of LHQW retrieved from TCMSP database, and successfully screened out 80 overlapping genes by intersecting with ALI disease target genes from OMIM and GeneCards databases. Among them, AKT1, TP53, IL6, VEGFA, TNF, JUN, STAT3, MAPK8, and MAPK1 were key genes with high degree value of >40. Akt1 is a family member of serine/threonine protein kinases that can reduce neutrophil recruitment and prevent ALI occurrence (Liu et al., 2013). Recent research has demonstrated that Akt1 is a key target for LHQW to treat COVID-19 (Xia et al., 2020). IL-6 and TNF-*α* are released by the activated neutrophils and macrophages, and are well recognized as pro-inflammatory cytokines and biomarkers for the diagnosis of sepsis (Aggarwal, 2003; Molano Franco et al., 2019). VEGFA, an isoform of the VEGF family, can induce physiological and pathological angiogenesis, which in turn promotes lung vessel permeability and aggravates LPS-induced ALI (Tang et al., 2016). Moreover, at the transcriptional level, Jun can enhance the production of inflammatory cytokines in sepsis-induced ALI (Cai et al., 2019), and STAT3 activated by IL-6 also contributes to the progression of ALI (Zhao et al., 2016). In addition to the key genes identified by PPI construction, GO analysis showed that apoptosis signaling pathways played a vital role in the biological processes of LHQW-ALI network. Compelling evidence shows that excessive apoptosis of alveolar epithelial cells exaggerates ALI (Galani et al., 2010; Hu et al., 2014), and inhibition of cell apoptosis effectively protects the lung tissues from ALI (Aggarwal et al., 2012). Apart from the regulation of inflammatory response, p53 mostly functions in acute injury by regulating apoptosis and cell arrest. In liver injury model, San huang yin chi decoction has been reported to improve hepatic injury by downregulating p53-mediated apoptosis signaling pathway (Diao et al., 2017). In acute lung injury, Physalis alkekengi L. var. franchetii can also alleviate LPS-induced ALI by targeting p53 and inhibiting cell apoptosis (Yang et al., 2020). Our study revealed that LPS increased the expression level of p53, activated the intrinsic apoptotosis pathways by upregulating Bax, caspase-3 and caspase-9 expression, and releasing cytochrome C from the mitochondria to the cytosol in the lung tissue of sepsis-induced ALI. Apart from the intrinsic apoptosis pathway, p53 could also mediate the extrinsic apoptosis pathway such as the activation of Fas (Nagata and Golstein, 1995), DR5 (Wu et al., 1997), and PERP (Singaravelu et al., 2009). A previous study has shown that Fas/FasL-dependent apoptosis also participates in the pathogenic mechanism of LPS-induced ALI (Kitamura et al., 2001). Thus, investigating the extrinsic apoptosis pathways regulated by p53 will allow a more comprehensive understanding of p53-mediated apoptosis in LPS-induced ALI. However, some reports have shown that p53 plays a protective role in ALI through the inhibition of inflammatory responses (Liu et al., 2009) and enhancement of endothelial barrier function (Barabutis et al., 2018). These studies are focusing on the inflammatory cells and endothelial cells. It is worth noting that different administration routes and dosage forms may lead to a discrepancy in results.

In addition, compared with pifithrin-*α*, medium- and high-dose LHQW displayed better effects on LPS-induced ALI, suggesting that the therapeutic effects of LHQW on sepsis-induced ALI are not limited to p53-mediated apoptosis pathway only. According to the KEGG results, several classical pathways, such as PI3K-Akt signaling pathway (Shi et al., 2019), NF-κB signaling pathway (Ju et al., 2018), cytokine-cytokine receptor interaction (Gouda and Bhandary, 2019), mitophagy-animal (Zhang et al., 2020), TGF-*β* signaling pathway (Liu et al., 2018) and complement and coagulation cascades (Chimenti et al., 2017), were regarded as the potential targets of LHQW for treating LPS-induced ALI.

Furthermore, quercetin, luteolin, kaempferol, and wogonin were identified as the active components of LHQW against LPS-induced ALI. All these natural active components are reported to exhibit antioxidative stress, reduce inflammatory cytokines, and inhibit neutrophil recruitment (Chen et al., 2012; Park et al., 2018; Wang et al., 2018), (Yao et al., 2014). This indicates that LHQW has the pharmacological component bases to attenuate LPS-induced ALI.

## Conclusion

In this study, a network pharmacology strategy was applied to predict the bioactive ingredients, effective targets, target genes, and signaling pathways of LHQW in the treatment of ALI. The significantly enriched biological process GO terms indicated that anti-apoptotic signaling molecules might be the most important pharmacological mechanism for LHQW in ALI. Moreover, we validated the results of network pharmacology approach in LPS-induced ALI mice treated with/without LHQW. Our findings revealed that LHQW was able to alleviate LPS-induced ALI *in vivo*. Furthermore, we observed that LHQW had protective effects on LPS-induced ALI *via* inhibiting p53-mediated intrinsic apoptotic pathways. In addition to p53-mediated apoptosis pathway, other signaling pathways may also contribute to the efficacy of LHQW, which await further studies.

## Data Availability

The original contributions presented in the study are included in the article/Supplementary Material, further inquiries can be directed to the corresponding author.

## Supplementary Material

Supplementary Figure S1 Herb-Component-Target Network of Lianhua Qingwen. Diamond nodes represent genes; circle nodes represent herbs; and the herb is named by acronym. Hexagon nodes of the same color that make a circle around herb represent components of the herb; and the components are named by acronym of corresponding to the herbs. Detailed information of the components are provided in Supplementary Table S1 **|** The remaining hexagon nodes represent a common component of two or more herbs. A, kaempferol; B, quercetin; C, beta-sitosterol; D1, sitosterol; D2, stigmasterol; E1, luteolin; E2, mairin; F1-F14, the common component of two herbs. The size of genes from large to small is arranged in descending order based on their degree values.

Supplementary Figure S2 The 11 Ingredient-80 overlapping genes PPI networks of **(A)** Lianqiao, **(B)** Jinyinhua, **(C)** Mahuang, **(D)** Kuxingren, **(E)** Banlangen, **(F)** Guanzhong, **(G)** Yuxingcao, **(H)** Guanghuoxiang, **(I)** Dahuang, **(J)** Hongjingtian, and **(K)** Gancao. Elliptical nodes delineate ingredients. Circle nodes represent components. They are named in the same way as those in Supplementary Figure S1. Diamond nodes delineate putative targets in 80 overlapping genes.
