# Supplementary material for: Mechanisms Underlying the Effects of Lianhua Qingwen on Sepsis-Induced Acute Lung Injury: A Network Pharmacology Approach
Source: Front Pharmacol. 2021 Oct 14;12:717652. doi: 10.3389/fphar.2021.717652 (PMC8551812; doi:10.3389/fphar.2021.717652)
Supplement: Supplementary file 1 [file Table1.DOCX]

Active compounds information of 11 herbs in LHQW

| Mol ID | Compound | OB(%) | DL | Medicine |
| --- | --- | --- | --- | --- |
| MOL000173 | wogonin | 30.68 | 0.23 | LQ |
| MOL003281 | 20(S)-dammar-24-ene-3β,20-diol-3-acetate | 40.23 | 0.82 | LQ |
| MOL003283 | (2R,3R,4S)-4-(4-hydroxy-3-methoxy-phenyl)-7-methoxy-2,3-dimethylol-tetralin-6-ol | 66.51 | 0.39 | LQ |
| MOL003290 | (3R,4R)-3,4-bis[(3,4-dimethoxyphenyl)methyl]oxolan-2-one | 52.3 | 0.48 | LQ |
| MOL003295 | (+)-pinoresinol monomethyl ether | 53.08 | 0.57 | LQ |
| MOL003305 | PHILLYRIN | 36.4 | 0.86 | LQ |
| MOL003306 | ACon1_001697 | 85.12 | 0.57 | LQ |
| MOL003308 | (+)-pinoresinol monomethyl ether-4-D-beta-glucoside_qt | 61.2 | 0.57 | LQ |
| MOL003315 | 3beta-Acetyl-20,25-epoxydammarane-24alpha-ol | 33.07 | 0.79 | LQ |
| MOL003322 | FORSYTHINOL | 81.25 | 0.57 | LQ |
| MOL003330 | (-)-Phillygenin | 95.04 | 0.57 | LQ |
| MOL003344 | β-amyrin acetate | 42.06 | 0.74 | LQ |
| MOL003347 | hyperforin | 44.03 | 0.6 | LQ |
| MOL003348 | adhyperforin | 44.03 | 0.61 | LQ |
| MOL003365 | Lactucasterol | 40.99 | 0.85 | LQ |
| MOL003370 | Onjixanthone I | 79.16 | 0.3 | LQ |
| MOL000358 | beta-sitosterol | 36.91 | 0.75 | LQ JYH MH BLG DH |
| MOL000422 | kaempferol | 41.88 | 0.24 | LQ JYH MH GZ YXC HJT GC |
| MOL000522 | arctiin | 34.45 | 0.84 | LQ |
| MOL000006 | luteolin | 36.16 | 0.25 | LQ JYH MH |
| MOL000791 | bicuculline | 69.67 | 0.88 | LQ |
| MOL000098 | quercetin | 46.43 | 0.28 | LQ JYH MH YXC GHX GC |
| MOL001494 | [Mandenol](https://tcmspw.com/molecule.php?qn=1494) | 42 | 0.19 | JYH MH |
| MOL001495 | [Ethyl linolenate](https://tcmspw.com/molecule.php?qn=1495) | 46.1 | 0.2 | JYH |
| MOL002707 | [phytofluene](https://tcmspw.com/molecule.php?qn=2707) | 43.18 | 0.5 | JYH |
| MOL002914 | [Eriodyctiol (flavanone)](https://tcmspw.com/molecule.php?qn=2914) | 41.35 | 0.24 | JYH |
| MOL003006 | [(-)-(3R,8S,9R,9aS,10aS)-9-ethenyl-8-(beta-D-glucopyranosyloxy)-2,3,9,9a,10,10a-hexahydro-5-oxo-5H,8H-pyrano[4,3-d]oxazolo[3,2-a]pyridine-3-carboxylic acid_qt](https://tcmspw.com/molecule.php?qn=3006) | 87.47 | 0.23 | JYH |
| MOL003014 | [secologanic dibutylacetal_qt](https://tcmspw.com/molecule.php?qn=3014) | 53.65 | 0.29 | JYH |
| MOL002773 | [beta-carotene](https://tcmspw.com/molecule.php?qn=2773) | 37.18 | 0.58 | JYH |
| MOL003036 | [ZINC03978781](https://tcmspw.com/molecule.php?qn=3036) | 43.83 | 0.76 | JYH |
| MOL003044 | [Chryseriol](https://tcmspw.com/molecule.php?qn=3044) | 35.85 | 0.27 | JYH |
| MOL003059 | [kryptoxanthin](https://tcmspw.com/molecule.php?qn=3059) | 47.25 | 0.57 | JYH |
| MOL003062 | [4,5'-Retro-.beta.,.beta.-Carotene-3,3'-dione, 4',5'-didehydro-](https://tcmspw.com/molecule.php?qn=3062) | 31.22 | 0.55 | JYH |
| MOL003095 | [5-hydroxy-7-methoxy-2-(3,4,5-trimethoxyphenyl)chromone](https://tcmspw.com/molecule.php?qn=3095) | 51.96 | 0.41 | JYH |
| MOL003101 | [7-epi-Vogeloside](https://tcmspw.com/molecule.php?qn=3101) | 46.13 | 0.58 | JYH |
| MOL003108 | [Caeruloside C](https://tcmspw.com/molecule.php?qn=3108) | 55.64 | 0.73 | JYH |
| MOL003111 | [Centauroside_qt](https://tcmspw.com/molecule.php?qn=3111) | 55.79 | 0.5 | JYH |
| MOL003117 | [Ioniceracetalides B_qt](https://tcmspw.com/molecule.php?qn=3117) | 61.19 | 0.19 | JYH |
| MOL003124 | [XYLOSTOSIDINE](https://tcmspw.com/molecule.php?qn=3124) | 43.17 | 0.64 | JYH |
| MOL003128 | [dinethylsecologanoside](https://tcmspw.com/molecule.php?qn=3128) | 48.46 | 0.48 | JYH |
| MOL000449 | [Stigmasterol](https://tcmspw.com/molecule.php?qn=449) | 43.83 | 0.76 | JYH KXR BLG MH |
| MOL010788 | [leucopelargonidin](https://tcmspw.com/molecule.php?qn=10788) | 57.97 | 0.24 | [MH](https://tcmspw.com/tcmspmol/MOL010788.mol2) |
| MOL002823 | [Herbacetin](https://tcmspw.com/molecule.php?qn=2823) | 36.07 | 0.27 | [MH](https://tcmspw.com/tcmspmol/MOL010788.mol2) |
| MOL010489 | [Resivit](https://tcmspw.com/molecule.php?qn=10489) | 30.84 | 0.27 | [MH](https://tcmspw.com/tcmspmol/MOL010788.mol2) |
| MOL004798 | [delphinidin](https://tcmspw.com/molecule.php?qn=4798) | 40.63 | 0.28 | [MH](https://tcmspw.com/tcmspmol/MOL010788.mol2) |
| MOL000492 | [(+)-catechin](https://tcmspw.com/molecule.php?qn=492) | 54.83 | 0.24 | [MH](https://tcmspw.com/tcmspmol/MOL010788.mol2) KXR |
| MOL001506 | [Supraene](https://tcmspw.com/molecule.php?qn=1506) | 33.55 | 0.42 | [MH](https://tcmspw.com/tcmspmol/MOL010788.mol2) |
| MOL001771 | [poriferast-5-en-3beta-ol](https://tcmspw.com/molecule.php?qn=1771) | 36.91 | 0.75 | [MH](https://tcmspw.com/tcmspmol/MOL010788.mol2) BLG |
| MOL002881 | [Diosmetin](https://tcmspw.com/molecule.php?qn=2881) | 31.14 | 0.27 | [MH](https://tcmspw.com/tcmspmol/MOL010788.mol2) |
| MOL004328 | [naringenin](https://tcmspw.com/molecule.php?qn=4328) | 59.29 | 0.21 | [MH](https://tcmspw.com/tcmspmol/MOL010788.mol2) GC |
| MOL004576 | [taxifolin](https://tcmspw.com/molecule.php?qn=4576) | 57.84 | 0.27 | [MH](https://tcmspw.com/tcmspmol/MOL010788.mol2) |
| MOL005043 | [campest-5-en-3beta-ol](https://tcmspw.com/molecule.php?qn=5043) | 37.58 | 0.71 | [MH](https://tcmspw.com/tcmspmol/MOL010788.mol2) |
| MOL005190 | [eriodictyol](https://tcmspw.com/molecule.php?qn=5190) | 71.79 | 0.24 | [MH](https://tcmspw.com/tcmspmol/MOL010788.mol2) |
| MOL005573 | [Genkwanin](https://tcmspw.com/molecule.php?qn=5573) | 37.13 | 0.24 | [MH](https://tcmspw.com/tcmspmol/MOL010788.mol2) GHX |
| MOL005842 | [Pectolinarigenin](https://tcmspw.com/molecule.php?qn=5842) | 41.17 | 0.3 | [MH](https://tcmspw.com/tcmspmol/MOL010788.mol2) |
| MOL007214 | [(+)-Leucocyanidin](https://tcmspw.com/molecule.php?qn=7214) | 37.61 | 0.27 | [MH](https://tcmspw.com/tcmspmol/MOL010788.mol2) |
| MOL011319 | [Truflex OBP](https://tcmspw.com/molecule.php?qn=11319) | 43.74 | 0.24 | [MH](https://tcmspw.com/tcmspmol/MOL010788.mol2) |
| MOL010921 | [estrone](https://tcmspw.com/molecule.php?qn=10921) | 53.56 | 0.32 | [KXR](https://tcmspw.com/tcmspmol/MOL010921.mol2) |
| MOL010922 | [Diisooctyl succinate](https://tcmspw.com/molecule.php?qn=10922) | 31.62 | 0.23 | [KXR](https://tcmspw.com/tcmspmol/MOL010921.mol2) |
| MOL002211 | [11,14-eicosadienoic acid](https://tcmspw.com/molecule.php?qn=2211) | 39.99 | 0.2 | [KXR](https://tcmspw.com/tcmspmol/MOL010921.mol2) |
| MOL002372 | [(6Z,10E,14E,18E)-2,6,10,15,19,23-hexamethyltetracosa-2,6,10,14,18,22-hexaene](https://tcmspw.com/molecule.php?qn=2372) | 33.55 | 0.42 | [KXR](https://tcmspw.com/tcmspmol/MOL010921.mol2) |
| MOL000359 | [sitosterol](https://tcmspw.com/molecule.php?qn=359) | 36.91 | 0.75 | [KXR](https://tcmspw.com/tcmspmol/MOL010921.mol2) BLG HJT GC |
| MOL005030 | [gondoic acid](https://tcmspw.com/molecule.php?qn=5030) | 30.7 | 0.2 | [KXR](https://tcmspw.com/tcmspmol/MOL010921.mol2) |
| MOL000953 | [CLR](https://tcmspw.com/molecule.php?qn=953) | 37.87 | 0.68 | [KXR](https://tcmspw.com/tcmspmol/MOL010921.mol2) BLG |
| MOL000211 | [Mairin](https://tcmspw.com/molecule.php?qn=211) | 55.38 | 0.78 | [KXR](https://tcmspw.com/tcmspmol/MOL010921.mol2) LQ GC |
| MOL002311 | [Glycyrol](https://tcmspw.com/molecule.php?qn=2311) | 90.78 | 0.67 | [KXR](https://tcmspw.com/tcmspmol/MOL010921.mol2) GC |
| MOL003410 | [Ziziphin_qt](https://tcmspw.com/molecule.php?qn=3410) | 66.95 | 0.62 | [KXR](https://tcmspw.com/tcmspmol/MOL010921.mol2) |
| MOL004355 | [Spinasterol](https://tcmspw.com/molecule.php?qn=4355) | 42.98 | 0.76 | [KXR](https://tcmspw.com/tcmspmol/MOL010921.mol2) YXC |
| MOL004841 | [Licochalcone B](https://tcmspw.com/molecule.php?qn=4841) | 76.76 | 0.19 | [KXR](https://tcmspw.com/tcmspmol/MOL010921.mol2) GC |
| MOL004903 | [liquiritin](https://tcmspw.com/molecule.php?qn=4903) | 65.69 | 0.74 | [KXR](https://tcmspw.com/tcmspmol/MOL010921.mol2) GC |
| MOL004908 | [Glabridin](https://tcmspw.com/molecule.php?qn=4908) | 53.25 | 0.47 | [KXR](https://tcmspw.com/tcmspmol/MOL010921.mol2) GC |
| MOL005017 | [Phaseol](https://tcmspw.com/molecule.php?qn=5017) | 78.77 | 0.58 | [KXR](https://tcmspw.com/tcmspmol/MOL010921.mol2) GC |
| MOL007207 | [Machiline](https://tcmspw.com/molecule.php?qn=7207) | 79.64 | 0.24 | [KXR](https://tcmspw.com/tcmspmol/MOL010921.mol2) |
| MOL012922 | [l-SPD](https://tcmspw.com/molecule.php?qn=12922) | 87.35 | 0.54 | [KXR](https://tcmspw.com/tcmspmol/MOL010921.mol2) |
| MOL001728 | [3-[ 2′ -( 5′ - hydroxymethyl) furyl ] -1 ( 2H) -isoquinolinone-7-O-BETA-D-glucoside_qt](https://tcmspw.com/molecule.php?qn=1728) | 51.74 | 0.18 | [BLG](https://tcmspw.com/tcmspmol/MOL001728.mol2) |
| MOL001792 | [DFV](https://tcmspw.com/molecule.php?qn=1792) | 32.76 | 0.18 | [BLG](https://tcmspw.com/tcmspmol/MOL001728.mol2) GC |
| MOL001756 | [quindoline](https://tcmspw.com/molecule.php?qn=1756) | 33.17 | 0.22 | [BLG](https://tcmspw.com/tcmspmol/MOL001728.mol2) |
| MOL001833 | [Glucobrassicin-1-Sulfonate_qt](https://tcmspw.com/molecule.php?qn=1833) | 42.52 | 0.24 | [BLG](https://tcmspw.com/tcmspmol/MOL001728.mol2) |
| MOL001689 | [acacetin](https://tcmspw.com/molecule.php?qn=1689) | 34.97 | 0.24 | [BLG](https://tcmspw.com/tcmspmol/MOL001728.mol2) |
| MOL001820 | [(E)-3-(3,5-dimethoxy-4-hydroxyb-enzylidene)-2-indolinone](https://tcmspw.com/molecule.php?qn=1820) | 65.17 | 0.25 | [BLG](https://tcmspw.com/tcmspmol/MOL001728.mol2) |
| MOL001814 | [(E)-3-(3,5-dimethoxy-4-hydroxy-benzylidene)-2-indolinone](https://tcmspw.com/molecule.php?qn=1814) | 57.18 | 0.25 | [BLG](https://tcmspw.com/tcmspmol/MOL001728.mol2) |
| MOL001782 | [(2Z)-2-(2-oxoindolin-3-ylidene)indolin-3-one](https://tcmspw.com/molecule.php?qn=1782) | 48.4 | 0.26 | [BLG](https://tcmspw.com/tcmspmol/MOL001728.mol2) |
| MOL001781 | [Indigo](https://tcmspw.com/molecule.php?qn=1781) | 38.2 | 0.26 | [BLG](https://tcmspw.com/tcmspmol/MOL001728.mol2) |
| MOL001735 | [Dinatin](https://tcmspw.com/molecule.php?qn=1735) | 30.97 | 0.27 | [BLG](https://tcmspw.com/tcmspmol/MOL001728.mol2) |
| MOL001798 | [neohesperidin_qt](https://tcmspw.com/molecule.php?qn=1798) | 71.17 | 0.27 | [BLG](https://tcmspw.com/tcmspmol/MOL001728.mol2) |
| MOL001736 | [(-)-taxifolin](https://tcmspw.com/molecule.php?qn=1736) | 60.51 | 0.27 | [BLG](https://tcmspw.com/tcmspmol/MOL001728.mol2) |
| MOL001767 | [hydroxyindirubin](https://tcmspw.com/molecule.php?qn=1767) | 63.37 | 0.3 | [BLG](https://tcmspw.com/tcmspmol/MOL001728.mol2) |
| MOL001774 | [Ineketone](https://tcmspw.com/molecule.php?qn=1774) | 37.14 | 0.3 | [BLG](https://tcmspw.com/tcmspmol/MOL001728.mol2) |
| MOL001722 | [2-O-beta-D-glucopyranosyl-2H-1,4-benzoxazin-3(4H)-one](https://tcmspw.com/molecule.php?qn=1722) | 43.62 | 0.31 | [BLG](https://tcmspw.com/tcmspmol/MOL001728.mol2) |
| MOL001793 | [(E)-2-[(3-indole)cyanomethylene-]-3-indolinone](https://tcmspw.com/molecule.php?qn=1793) | 54.59 | 0.32 | [BLG](https://tcmspw.com/tcmspmol/MOL001728.mol2) |
| MOL001749 | [ZINC03860434](https://tcmspw.com/molecule.php?qn=1749) | 43.59 | 0.35 | [BLG](https://tcmspw.com/tcmspmol/MOL001728.mol2) |
| MOL001733 | [EUPATORIN](https://tcmspw.com/molecule.php?qn=1733) | 30.23 | 0.37 | [BLG](https://tcmspw.com/tcmspmol/MOL001728.mol2) |
| MOL001721 | [Isaindigodione](https://tcmspw.com/molecule.php?qn=1721) | 60.12 | 0.41 | [BLG](https://tcmspw.com/tcmspmol/MOL001728.mol2) |
| MOL001803 | [Sinensetin](https://tcmspw.com/molecule.php?qn=1803) | 50.56 | 0.45 | [BLG](https://tcmspw.com/tcmspmol/MOL001728.mol2) |
| MOL001779 | [Sinoacutine](https://tcmspw.com/molecule.php?qn=1779) | 49.11 | 0.46 | [BLG](https://tcmspw.com/tcmspmol/MOL001728.mol2) |
| MOL001734 | [3-[[(2R,3R,5R,6S)-3,5-dihydroxy-6-(1H-indol-3-yloxy)-4-oxooxan-2-yl]methoxy]-3-oxopropanoic acid](https://tcmspw.com/molecule.php?qn=1734) | 85.87 | 0.47 | [BLG](https://tcmspw.com/tcmspmol/MOL001728.mol2) |
| MOL001750 | [glucobrassicin](https://tcmspw.com/molecule.php?qn=1750) | 66.02 | 0.48 | [BLG](https://tcmspw.com/tcmspmol/MOL001728.mol2) |
| MOL001726 | [pinoresinol-4-O-beta-D-apiosyl-beta-D-glucopyranoside](https://tcmspw.com/molecule.php?qn=1726) | 36.45 | 0.51 | [BLG](https://tcmspw.com/tcmspmol/MOL001728.mol2) |
| MOL001828 | [3-[(3,5-dimethoxy-4-oxo-1-cyclohexa-2,5-dienylidene)methyl]-2,4-dihydro-1H-pyrrolo[2,1-b]quinazolin-9-one](https://tcmspw.com/molecule.php?qn=1828) | 51.84 | 0.56 | [BLG](https://tcmspw.com/tcmspmol/MOL001728.mol2) |
| MOL001769 | [beta-sitosterol dodecantate](https://tcmspw.com/molecule.php?qn=1769) | 34.57 | 0.57 | [BLG](https://tcmspw.com/tcmspmol/MOL001728.mol2) |
| MOL001783 | [2-(9-((3-methyl-2-oxopent-3-en-1-yl)oxy)-2-oxo-1,2,8,9-tetrahydrofuro[2,3-h]quinolin-8-yl)propan-2-yl acetate](https://tcmspw.com/molecule.php?qn=1783) | 64 | 0.57 | [BLG](https://tcmspw.com/tcmspmol/MOL001728.mol2) |
| MOL001790 | [Linarin](https://tcmspw.com/molecule.php?qn=1790) | 39.84 | 0.71 | [BLG](https://tcmspw.com/tcmspmol/MOL001728.mol2) |
| MOL001800 | [rosasterol](https://tcmspw.com/molecule.php?qn=1800) | 35.87 | 0.75 | [BLG](https://tcmspw.com/tcmspmol/MOL001728.mol2) |
| MOL001755 | [24-Ethylcholest-4-en-3-one](https://tcmspw.com/molecule.php?qn=1755) | 36.08 | 0.76 | [BLG](https://tcmspw.com/tcmspmol/MOL001728.mol2) MH |
| MOL001804 | [Stigmasta-5,22-diene-3beta,7alpha-diol](https://tcmspw.com/molecule.php?qn=1804) | 43.04 | 0.82 | [BLG](https://tcmspw.com/tcmspmol/MOL001728.mol2) |
| MOL001806 | [Stigmasta-5,22-diene-3beta,7beta-diol](https://tcmspw.com/molecule.php?qn=1806) | 42.56 | 0.83 | [BLG](https://tcmspw.com/tcmspmol/MOL001728.mol2) |
| MOL001810 | [6-(3-oxoindolin-2-ylidene)indolo[2,1-b]quinazolin-12-one](https://tcmspw.com/molecule.php?qn=1810) | 45.28 | 0.89 | [BLG](https://tcmspw.com/tcmspmol/MOL001728.mol2) |
| MOL001040 | [(2R)-5,7-dihydroxy-2-(4-hydroxyphenyl)chroman-4-one](https://tcmspw.com/molecule.php?qn=1040) | 42.36 | 0.21 | [GZ](https://tcmspw.com/tcmspmol/MOL001040.mol2) |
| MOL002605 | [11-Hydroxynumantenine](https://tcmspw.com/molecule.php?qn=2605) | 50.79 | 0.71 | [GZ](https://tcmspw.com/tcmspmol/MOL001040.mol2) |
| MOL002609 | [Harmonyl](https://tcmspw.com/molecule.php?qn=2609) | 43.8 | 0.46 | [GZ](https://tcmspw.com/tcmspmol/MOL001040.mol2) |
| MOL002610 | [ZINC00035529](https://tcmspw.com/molecule.php?qn=2610) | 58.39 | 0.22 | [GZ](https://tcmspw.com/tcmspmol/MOL001040.mol2) |
| MOL002614 | [Flavidin](https://tcmspw.com/molecule.php?qn=2614) | 30.1 | 0.26 | [GZ](https://tcmspw.com/tcmspmol/MOL001040.mol2) |
| MOL002619 | [Albaspidin AA](https://tcmspw.com/molecule.php?qn=2619) | 31.16 | 0.36 | [GZ](https://tcmspw.com/tcmspmol/MOL001040.mol2) |
| MOL003851 | [Isoramanone](https://tcmspw.com/molecule.php?qn=3851) | 39.97 | 0.51 | [YXC](https://tcmspw.com/tcmspmol/MOL003851.mol2) |
| MOL004345 | [1-methyl-2-nonacosyl-4-quinolone](https://tcmspw.com/molecule.php?qn=4345) | 31.54 | 0.5 | [YXC](https://tcmspw.com/tcmspmol/MOL003851.mol2) |
| MOL004350 | [Ruvoside_qt](https://tcmspw.com/molecule.php?qn=4350) | 36.12 | 0.76 | [YXC](https://tcmspw.com/tcmspmol/MOL003851.mol2) |
| MOL004351 | [C09747](https://tcmspw.com/molecule.php?qn=4351) | 37.28 | 0.25 | [YXC](https://tcmspw.com/tcmspmol/MOL003851.mol2) |
| MOL002879 | [Diop](https://tcmspw.com/molecule.php?qn=2879) | 43.59 | 0.39 | [GHX](https://tcmspw.com/tcmspmol/MOL002879.mol2) |
| MOL005884 | [patchoulan 1,12-diol](https://tcmspw.com/molecule.php?qn=5884) | 38.17 | 0.25 | [GHX](https://tcmspw.com/tcmspmol/MOL002879.mol2) |
| MOL005890 | [pachypodol](https://tcmspw.com/molecule.php?qn=5890) | 75.06 | 0.4 | [GHX](https://tcmspw.com/tcmspmol/MOL002879.mol2) |
| MOL005911 | [5-Hydroxy-7,4'-dimethoxyflavanon](https://tcmspw.com/molecule.php?qn=5911) | 51.54 | 0.27 | [GHX](https://tcmspw.com/tcmspmol/MOL002879.mol2) |
| MOL005916 | [irisolidone](https://tcmspw.com/molecule.php?qn=5916) | 37.78 | 0.3 | [GHX](https://tcmspw.com/tcmspmol/MOL002879.mol2) |
| MOL005918 | [phenanthrone](https://tcmspw.com/molecule.php?qn=5918) | 38.7 | 0.33 | [GHX](https://tcmspw.com/tcmspmol/MOL002879.mol2) |
| MOL005921 | [quercetin 7-O-β-D-glucoside](https://tcmspw.com/molecule.php?qn=5921) | 49.57 | 0.27 | [GHX](https://tcmspw.com/tcmspmol/MOL002879.mol2) |
| MOL005922 | [Acanthoside B](https://tcmspw.com/molecule.php?qn=5922) | 43.35 | 0.77 | [GHX](https://tcmspw.com/tcmspmol/MOL002879.mol2) |
| MOL005923 | [3,23-dihydroxy-12-oleanen-28-oic acid](https://tcmspw.com/molecule.php?qn=5923) | 30.86 | 0.86 | [GHX](https://tcmspw.com/tcmspmol/MOL002879.mol2) |
| MOL002235 | [EUPATIN](https://tcmspw.com/molecule.php?qn=2235) | 50.8 | 0.41 | [DH](https://tcmspw.com/tcmspmol/MOL002235.mol2) |
| MOL002251 | [Mutatochrome](https://tcmspw.com/molecule.php?qn=2251) | 48.64 | 0.61 | [DH](https://tcmspw.com/tcmspmol/MOL002235.mol2) |
| MOL002259 | [Physciondiglucoside](https://tcmspw.com/molecule.php?qn=2259) | 41.65 | 0.63 | [DH](https://tcmspw.com/tcmspmol/MOL002235.mol2) |
| MOL002260 | [Procyanidin B-5,3'-O-gallate](https://tcmspw.com/molecule.php?qn=2260) | 31.99 | 0.32 | [DH](https://tcmspw.com/tcmspmol/MOL002235.mol2) |
| MOL002268 | [rhein](https://tcmspw.com/molecule.php?qn=2268) | 47.07 | 0.28 | [DH](https://tcmspw.com/tcmspmol/MOL002235.mol2) |
| MOL002276 | [Sennoside E_qt](https://tcmspw.com/molecule.php?qn=2276) | 50.69 | 0.61 | [DH](https://tcmspw.com/tcmspmol/MOL002235.mol2) |
| MOL002280 | [Torachrysone-8-O-beta-D-(6'-oxayl)-glucoside](https://tcmspw.com/molecule.php?qn=2280) | 43.02 | 0.74 | [DH](https://tcmspw.com/tcmspmol/MOL002235.mol2) |
| MOL002281 | [Toralactone](https://tcmspw.com/molecule.php?qn=2281) | 46.46 | 0.24 | [DH](https://tcmspw.com/tcmspmol/MOL002235.mol2) |
| MOL002288 | [Emodin-1-O-beta-D-glucopyranoside](https://tcmspw.com/molecule.php?qn=2288) | 44.81 | 0.8 | [DH](https://tcmspw.com/tcmspmol/MOL002235.mol2) |
| MOL002293 | [Sennoside D_qt](https://tcmspw.com/molecule.php?qn=2293) | 61.06 | 0.61 | [DH](https://tcmspw.com/tcmspmol/MOL002235.mol2) |
| MOL002297 | [Daucosterol_qt](https://tcmspw.com/molecule.php?qn=2297) | 35.89 | 0.7 | [DH](https://tcmspw.com/tcmspmol/MOL002235.mol2) |
| MOL002303 | [palmidin A](https://tcmspw.com/molecule.php?qn=2303) | 32.45 | 0.65 | [DH](https://tcmspw.com/tcmspmol/MOL002235.mol2) |
| MOL000471 | [aloe-emodin](https://tcmspw.com/molecule.php?qn=471) | 83.38 | 0.24 | [DH](https://tcmspw.com/tcmspmol/MOL002235.mol2) |
| MOL000554 | [gallic acid-3-O-(6'-O-galloyl)-glucoside](https://tcmspw.com/molecule.php?qn=554) | 30.25 | 0.67 | [DH](https://tcmspw.com/tcmspmol/MOL002235.mol2) |
| MOL000096 | [(-)-catechin](https://tcmspw.com/molecule.php?qn=96) | 49.68 | 0.24 | [DH](https://tcmspw.com/tcmspmol/MOL002235.mol2) |
| MOL003341 | salidroside | 15.96 | 0.2 | HJT |
| MOL001484 | [Inermine](https://tcmspw.com/molecule.php?qn=1484) | 75.18 | 0.54 | [GC](https://tcmspw.com/tcmspmol/MOL001484.mol2) |
| MOL000239 | [Jaranol](https://tcmspw.com/molecule.php?qn=239) | 50.83 | 0.29 | [GC](https://tcmspw.com/tcmspmol/MOL001484.mol2) |
| MOL002565 | [Medicarpin](https://tcmspw.com/molecule.php?qn=2565) | 49.22 | 0.34 | [GC](https://tcmspw.com/tcmspmol/MOL001484.mol2) |
| MOL000354 | [isorhamnetin](https://tcmspw.com/molecule.php?qn=354) | 49.6 | 0.31 | [GC](https://tcmspw.com/tcmspmol/MOL001484.mol2) |
| MOL003656 | [Lupiwighteone](https://tcmspw.com/molecule.php?qn=3656) | 51.64 | 0.37 | [GC](https://tcmspw.com/tcmspmol/MOL001484.mol2) |
| MOL003896 | [7-Methoxy-2-methyl isoflavone](https://tcmspw.com/molecule.php?qn=3896) | 42.56 | 0.2 | [GC](https://tcmspw.com/tcmspmol/MOL001484.mol2) |
| MOL000392 | [formononetin](https://tcmspw.com/molecule.php?qn=392) | 69.67 | 0.21 | [GC](https://tcmspw.com/tcmspmol/MOL001484.mol2) |
| MOL000417 | [Calycosin](https://tcmspw.com/molecule.php?qn=417) | 47.75 | 0.24 | [GC](https://tcmspw.com/tcmspmol/MOL001484.mol2) |
| MOL004805 | [(2S)-2-[4-hydroxy-3-(3-methylbut-2-enyl)phenyl]-8,8-dimethyl-2,3-dihydropyrano[2,3-f]chromen-4-one](https://tcmspw.com/molecule.php?qn=4805) | 31.79 | 0.72 | [GC](https://tcmspw.com/tcmspmol/MOL001484.mol2) |
| MOL004806 | [euchrenone](https://tcmspw.com/molecule.php?qn=4806) | 30.29 | 0.57 | [GC](https://tcmspw.com/tcmspmol/MOL001484.mol2) |
| MOL004808 | [glyasperin B](https://tcmspw.com/molecule.php?qn=4808) | 65.22 | 0.44 | [GC](https://tcmspw.com/tcmspmol/MOL001484.mol2) |
| MOL004810 | [glyasperin F](https://tcmspw.com/molecule.php?qn=4810) | 75.84 | 0.54 | [GC](https://tcmspw.com/tcmspmol/MOL001484.mol2) |
| MOL004811 | [Glyasperin C](https://tcmspw.com/molecule.php?qn=4811) | 45.56 | 0.4 | [GC](https://tcmspw.com/tcmspmol/MOL001484.mol2) |
| MOL004814 | [Isotrifoliol](https://tcmspw.com/molecule.php?qn=4814) | 31.94 | 0.42 | [GC](https://tcmspw.com/tcmspmol/MOL001484.mol2) |
| MOL004815 | [(E)-1-(2,4-dihydroxyphenyl)-3-(2,2-dimethylchromen-6-yl)prop-2-en-1-one](https://tcmspw.com/molecule.php?qn=4815) | 39.62 | 0.35 | [GC](https://tcmspw.com/tcmspmol/MOL001484.mol2) |
| MOL004820 | [kanzonols W](https://tcmspw.com/molecule.php?qn=4820) | 50.48 | 0.52 | [GC](https://tcmspw.com/tcmspmol/MOL001484.mol2) |
| MOL004824 | [(2S)-6-(2,4-dihydroxyphenyl)-2-(2-hydroxypropan-2-yl)-4-methoxy-2,3-dihydrofuro[3,2-g]chromen-7-one](https://tcmspw.com/molecule.php?qn=4824) | 60.25 | 0.63 | [GC](https://tcmspw.com/tcmspmol/MOL001484.mol2) |
| MOL004827 | [Semilicoisoflavone B](https://tcmspw.com/molecule.php?qn=4827) | 48.78 | 0.55 | [GC](https://tcmspw.com/tcmspmol/MOL001484.mol2) |
| MOL004828 | [Glepidotin A](https://tcmspw.com/molecule.php?qn=4828) | 44.72 | 0.35 | [GC](https://tcmspw.com/tcmspmol/MOL001484.mol2) |
| MOL004829 | [Glepidotin B](https://tcmspw.com/molecule.php?qn=4829) | 64.46 | 0.34 | [GC](https://tcmspw.com/tcmspmol/MOL001484.mol2) |
| MOL004833 | [Phaseolinisoflavan](https://tcmspw.com/molecule.php?qn=4833) | 32.01 | 0.45 | [GC](https://tcmspw.com/tcmspmol/MOL001484.mol2) |
| MOL004835 | [Glypallichalcone](https://tcmspw.com/molecule.php?qn=4835) | 61.6 | 0.19 | [GC](https://tcmspw.com/tcmspmol/MOL001484.mol2) |
| MOL004838 | [8-(6-hydroxy-2-benzofuranyl)-2,2-dimethyl-5-chromenol](https://tcmspw.com/molecule.php?qn=4838) | 58.44 | 0.38 | [GC](https://tcmspw.com/tcmspmol/MOL001484.mol2) |
| MOL004848 | [licochalcone G](https://tcmspw.com/molecule.php?qn=4848) | 49.25 | 0.32 | [GC](https://tcmspw.com/tcmspmol/MOL001484.mol2) |
| MOL004849 | [3-(2,4-dihydroxyphenyl)-8-(1,1-dimethylprop-2-enyl)-7-hydroxy-5-methoxy-coumarin](https://tcmspw.com/molecule.php?qn=4849) | 59.62 | 0.43 | [GC](https://tcmspw.com/tcmspmol/MOL001484.mol2) |
| MOL004855 | [Licoricone](https://tcmspw.com/molecule.php?qn=4855) | 63.58 | 0.47 | [GC](https://tcmspw.com/tcmspmol/MOL001484.mol2) |
| MOL004856 | [Gancaonin A](https://tcmspw.com/molecule.php?qn=4856) | 51.08 | 0.4 | [GC](https://tcmspw.com/tcmspmol/MOL001484.mol2) |
| MOL004857 | [Gancaonin B](https://tcmspw.com/molecule.php?qn=4857) | 48.79 | 0.45 | [GC](https://tcmspw.com/tcmspmol/MOL001484.mol2) |
| MOL004860 | [licorice glycoside E](https://tcmspw.com/molecule.php?qn=4860) | 32.89 | 0.27 | [GC](https://tcmspw.com/tcmspmol/MOL001484.mol2) |
| MOL004863 | [3-(3,4-dihydroxyphenyl)-5,7-dihydroxy-8-(3-methylbut-2-enyl)chromone](https://tcmspw.com/molecule.php?qn=4863) | 66.37 | 0.41 | [GC](https://tcmspw.com/tcmspmol/MOL001484.mol2) |
| MOL004864 | [5,7-dihydroxy-3-(4-methoxyphenyl)-8-(3-methylbut-2-enyl)chromone](https://tcmspw.com/molecule.php?qn=4864) | 30.49 | 0.41 | [GC](https://tcmspw.com/tcmspmol/MOL001484.mol2) |
| MOL004866 | [2-(3,4-dihydroxyphenyl)-5,7-dihydroxy-6-(3-methylbut-2-enyl)chromone](https://tcmspw.com/molecule.php?qn=4866) | 44.15 | 0.41 | [GC](https://tcmspw.com/tcmspmol/MOL001484.mol2) |
| MOL004879 | [Glycyrin](https://tcmspw.com/molecule.php?qn=4879) | 52.61 | 0.47 | [GC](https://tcmspw.com/tcmspmol/MOL001484.mol2) |
| MOL004882 | [Licocoumarone](https://tcmspw.com/molecule.php?qn=4882) | 33.21 | 0.36 | [GC](https://tcmspw.com/tcmspmol/MOL001484.mol2) |
| MOL004883 | [Licoisoflavone](https://tcmspw.com/molecule.php?qn=4883) | 41.61 | 0.42 | [GC](https://tcmspw.com/tcmspmol/MOL001484.mol2) |
| MOL004884 | [Licoisoflavone B](https://tcmspw.com/molecule.php?qn=4884) | 38.93 | 0.55 | [GC](https://tcmspw.com/tcmspmol/MOL001484.mol2) |
| MOL004885 | [licoisoflavanone](https://tcmspw.com/molecule.php?qn=4885) | 52.47 | 0.54 | [GC](https://tcmspw.com/tcmspmol/MOL001484.mol2) |
| MOL004891 | [shinpterocarpin](https://tcmspw.com/molecule.php?qn=4891) | 80.3 | 0.73 | [GC](https://tcmspw.com/tcmspmol/MOL001484.mol2) |
| MOL004898 | [(E)-3-[3,4-dihydroxy-5-(3-methylbut-2-enyl)phenyl]-1-(2,4-dihydroxyphenyl)prop-2-en-1-one](https://tcmspw.com/molecule.php?qn=4898) | 46.27 | 0.31 | [GC](https://tcmspw.com/tcmspmol/MOL001484.mol2) |
| MOL004904 | [licopyranocoumarin](https://tcmspw.com/molecule.php?qn=4904) | 80.36 | 0.65 | [GC](https://tcmspw.com/tcmspmol/MOL001484.mol2) |
| MOL004905 | [3,22-Dihydroxy-11-oxo-delta(12)-oleanene-27-alpha-methoxycarbonyl-29-oic acid](https://tcmspw.com/molecule.php?qn=4905) | 34.32 | 0.55 | [GC](https://tcmspw.com/tcmspmol/MOL001484.mol2) |
| MOL004907 | [Glyzaglabrin](https://tcmspw.com/molecule.php?qn=4907) | 61.07 | 0.35 | [GC](https://tcmspw.com/tcmspmol/MOL001484.mol2) |
| MOL004910 | [Glabranin](https://tcmspw.com/molecule.php?qn=4910) | 52.9 | 0.31 | [GC](https://tcmspw.com/tcmspmol/MOL001484.mol2) |
| MOL004911 | [Glabrene](https://tcmspw.com/molecule.php?qn=4911) | 46.27 | 0.44 | [GC](https://tcmspw.com/tcmspmol/MOL001484.mol2) |
| MOL004912 | [Glabrone](https://tcmspw.com/molecule.php?qn=4912) | 52.51 | 0.5 | [GC](https://tcmspw.com/tcmspmol/MOL001484.mol2) |
| MOL004913 | [1,3-dihydroxy-9-methoxy-6-benzofurano[3,2-c]chromenone](https://tcmspw.com/molecule.php?qn=4913) | 48.14 | 0.43 | [GC](https://tcmspw.com/tcmspmol/MOL001484.mol2) |
| MOL004914 | [1,3-dihydroxy-8,9-dimethoxy-6-benzofurano[3,2-c]chromenone](https://tcmspw.com/molecule.php?qn=4914) | 62.9 | 0.53 | [GC](https://tcmspw.com/tcmspmol/MOL001484.mol2) |
| MOL004915 | [Eurycarpin A](https://tcmspw.com/molecule.php?qn=4915) | 43.28 | 0.37 | [GC](https://tcmspw.com/tcmspmol/MOL001484.mol2) |
| MOL004917 | [glycyroside](https://tcmspw.com/molecule.php?qn=4917) | 37.25 | 0.79 | [GC](https://tcmspw.com/tcmspmol/MOL001484.mol2) |
| MOL004924 | [(-)-Medicocarpin](https://tcmspw.com/molecule.php?qn=4924) | 40.99 | 0.95 | [GC](https://tcmspw.com/tcmspmol/MOL001484.mol2) |
| MOL004935 | [Sigmoidin-B](https://tcmspw.com/molecule.php?qn=4935) | 34.88 | 0.41 | [GC](https://tcmspw.com/tcmspmol/MOL001484.mol2) |
| MOL004941 | [(2R)-7-hydroxy-2-(4-hydroxyphenyl)chroman-4-one](https://tcmspw.com/molecule.php?qn=4941) | 71.12 | 0.18 | [GC](https://tcmspw.com/tcmspmol/MOL001484.mol2) |
| MOL004945 | [(2S)-7-hydroxy-2-(4-hydroxyphenyl)-8-(3-methylbut-2-enyl)chroman-4-one](https://tcmspw.com/molecule.php?qn=4945) | 36.57 | 0.32 | [GC](https://tcmspw.com/tcmspmol/MOL001484.mol2) |
| MOL004948 | [Isoglycyrol](https://tcmspw.com/molecule.php?qn=4948) | 44.7 | 0.84 | [GC](https://tcmspw.com/tcmspmol/MOL001484.mol2) |
| MOL004949 | [Isolicoflavonol](https://tcmspw.com/molecule.php?qn=4949) | 45.17 | 0.42 | [GC](https://tcmspw.com/tcmspmol/MOL001484.mol2) |
| MOL004957 | [HMO](https://tcmspw.com/molecule.php?qn=4957) | 38.37 | 0.21 | [GC](https://tcmspw.com/tcmspmol/MOL001484.mol2) |
| MOL004959 | [1-Methoxyphaseollidin](https://tcmspw.com/molecule.php?qn=4959) | 69.98 | 0.64 | [GC](https://tcmspw.com/tcmspmol/MOL001484.mol2) |
| MOL004961 | [Quercetin der.](https://tcmspw.com/molecule.php?qn=4961) | 46.45 | 0.33 | [GC](https://tcmspw.com/tcmspmol/MOL001484.mol2) |
| MOL004966 | [3'-Hydroxy-4'-O-Methylglabridin](https://tcmspw.com/molecule.php?qn=4966) | 43.71 | 0.57 | [GC](https://tcmspw.com/tcmspmol/MOL001484.mol2) |
| MOL000497 | [licochalcone a](https://tcmspw.com/molecule.php?qn=497) | 40.79 | 0.29 | [GC](https://tcmspw.com/tcmspmol/MOL001484.mol2) |
| MOL004974 | [3'-Methoxyglabridin](https://tcmspw.com/molecule.php?qn=4974) | 46.16 | 0.57 | [GC](https://tcmspw.com/tcmspmol/MOL001484.mol2) |
| MOL004978 | [2-[(3R)-8,8-dimethyl-3,4-dihydro-2H-pyrano[6,5-f]chromen-3-yl]-5-methoxyphenol](https://tcmspw.com/molecule.php?qn=4978) | 36.21 | 0.52 | [GC](https://tcmspw.com/tcmspmol/MOL001484.mol2) |
| MOL004980 | [Inflacoumarin A](https://tcmspw.com/molecule.php?qn=4980) | 39.71 | 0.33 | [GC](https://tcmspw.com/tcmspmol/MOL001484.mol2) |
| MOL004985 | [icos-5-enoic acid](https://tcmspw.com/molecule.php?qn=4985) | 30.7 | 0.2 | [GC](https://tcmspw.com/tcmspmol/MOL001484.mol2) |
| MOL004988 | [Kanzonol F](https://tcmspw.com/molecule.php?qn=4988) | 32.47 | 0.89 | [GC](https://tcmspw.com/tcmspmol/MOL001484.mol2) |
| MOL004989 | [6-prenylated eriodictyol](https://tcmspw.com/molecule.php?qn=4989) | 39.22 | 0.41 | [GC](https://tcmspw.com/tcmspmol/MOL001484.mol2) |
| MOL004990 | [7,2',4'-trihydroxy－5-methoxy-3－arylcoumarin](https://tcmspw.com/molecule.php?qn=4990) | 83.71 | 0.27 | [GC](https://tcmspw.com/tcmspmol/MOL001484.mol2) |
| MOL004991 | [7-Acetoxy-2-methylisoflavone](https://tcmspw.com/molecule.php?qn=4991) | 38.92 | 0.26 | [GC](https://tcmspw.com/tcmspmol/MOL001484.mol2) |
| MOL004993 | [8-prenylated eriodictyol](https://tcmspw.com/molecule.php?qn=4993) | 53.79 | 0.4 | [GC](https://tcmspw.com/tcmspmol/MOL001484.mol2) |
| MOL004996 | [gadelaidic acid](https://tcmspw.com/molecule.php?qn=4996) | 30.7 | 0.2 | [GC](https://tcmspw.com/tcmspmol/MOL001484.mol2) |
| MOL000500 | [Vestitol](https://tcmspw.com/molecule.php?qn=500) | 74.66 | 0.21 | [GC](https://tcmspw.com/tcmspmol/MOL001484.mol2) |
| MOL005000 | [Gancaonin G](https://tcmspw.com/molecule.php?qn=5000) | 60.44 | 0.39 | [GC](https://tcmspw.com/tcmspmol/MOL001484.mol2) |
| MOL005001 | [Gancaonin H](https://tcmspw.com/molecule.php?qn=5001) | 50.1 | 0.78 | [GC](https://tcmspw.com/tcmspmol/MOL001484.mol2) |
| MOL005003 | [Licoagrocarpin](https://tcmspw.com/molecule.php?qn=5003) | 58.81 | 0.58 | [GC](https://tcmspw.com/tcmspmol/MOL001484.mol2) |
| MOL005007 | [Glyasperins M](https://tcmspw.com/molecule.php?qn=5007) | 72.67 | 0.59 | [GC](https://tcmspw.com/tcmspmol/MOL001484.mol2) |
| MOL005008 | [Glycyrrhiza flavonol A](https://tcmspw.com/molecule.php?qn=5008) | 41.28 | 0.6 | [GC](https://tcmspw.com/tcmspmol/MOL001484.mol2) |
| MOL005012 | [Licoagroisoflavone](https://tcmspw.com/molecule.php?qn=5012) | 57.28 | 0.49 | [GC](https://tcmspw.com/tcmspmol/MOL001484.mol2) |
| MOL005013 | [18α-hydroxyglycyrrhetic acid](https://tcmspw.com/molecule.php?qn=5013) | 41.16 | 0.71 | [GC](https://tcmspw.com/tcmspmol/MOL001484.mol2) |
| MOL005016 | [Odoratin](https://tcmspw.com/molecule.php?qn=5016) | 49.95 | 0.3 | [GC](https://tcmspw.com/tcmspmol/MOL001484.mol2) |
| MOL005018 | [Xambioona](https://tcmspw.com/molecule.php?qn=5018) | 54.85 | 0.87 | [GC](https://tcmspw.com/tcmspmol/MOL001484.mol2) |
| MOL005020 | [dehydroglyasperins C](https://tcmspw.com/molecule.php?qn=5020) | 53.82 | 0.37 | [GC](https://tcmspw.com/tcmspmol/MOL001484.mol2) |
