# Supplementary figures and images for: Mechanisms Underlying the Effects of Lianhua Qingwen on Sepsis-Induced Acute Lung Injury: A Network Pharmacology Approach
Source: Front Pharmacol. 2021 Oct 14;12:717652. doi: 10.3389/fphar.2021.717652 (PMC8551812; doi:10.3389/fphar.2021.717652)

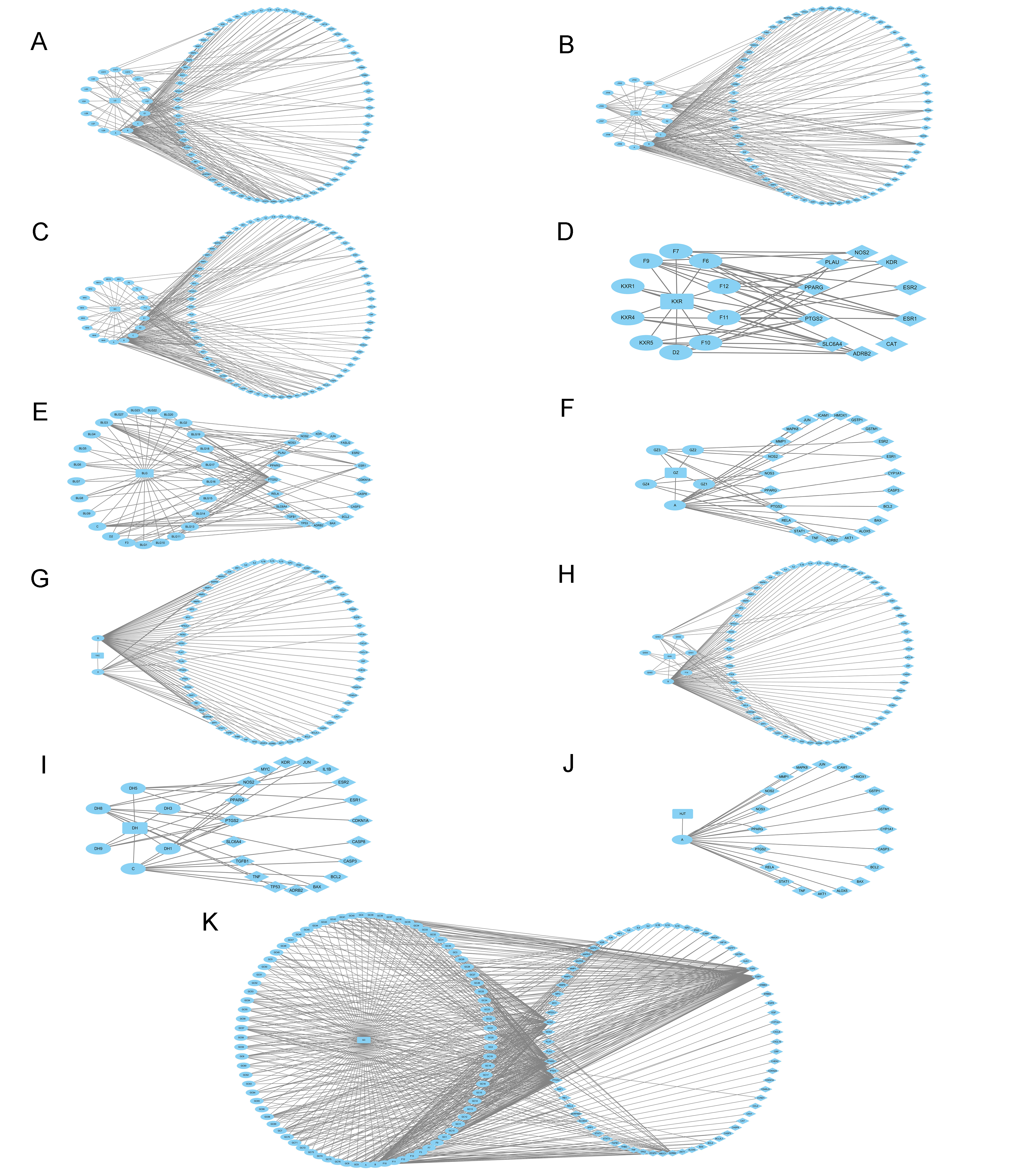

Supplement: Supplementary file 2 [file Image2.TIF]

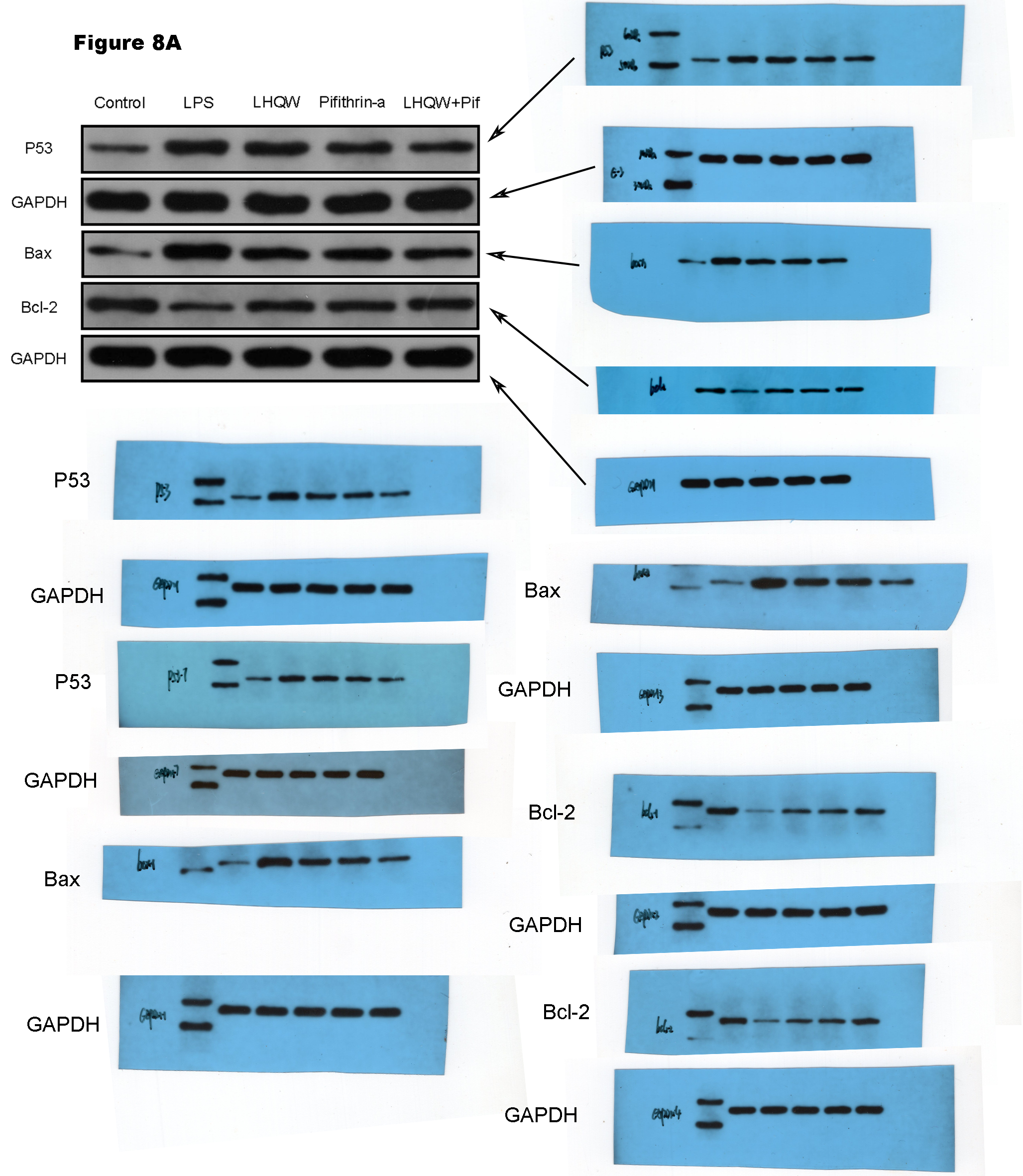


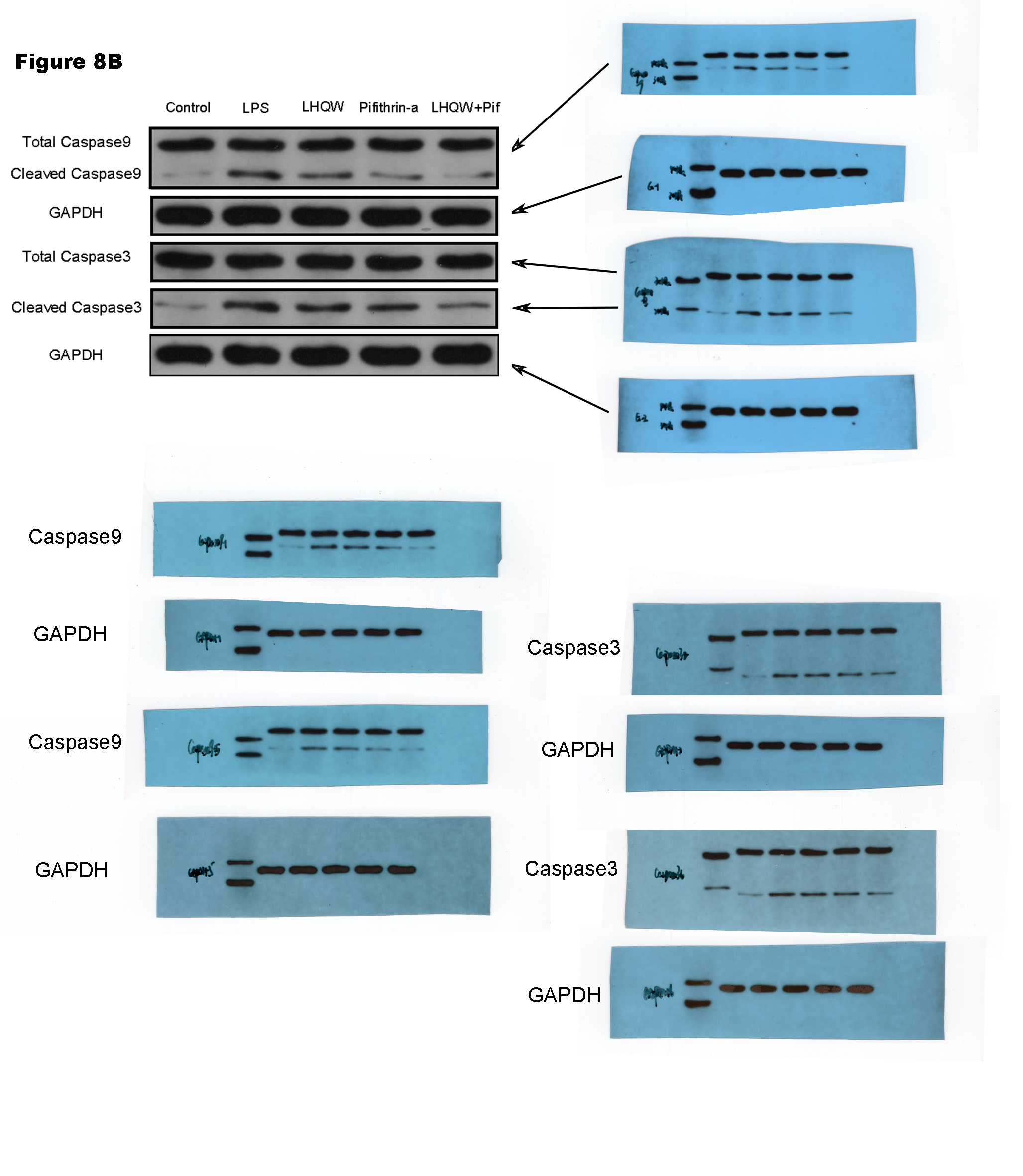


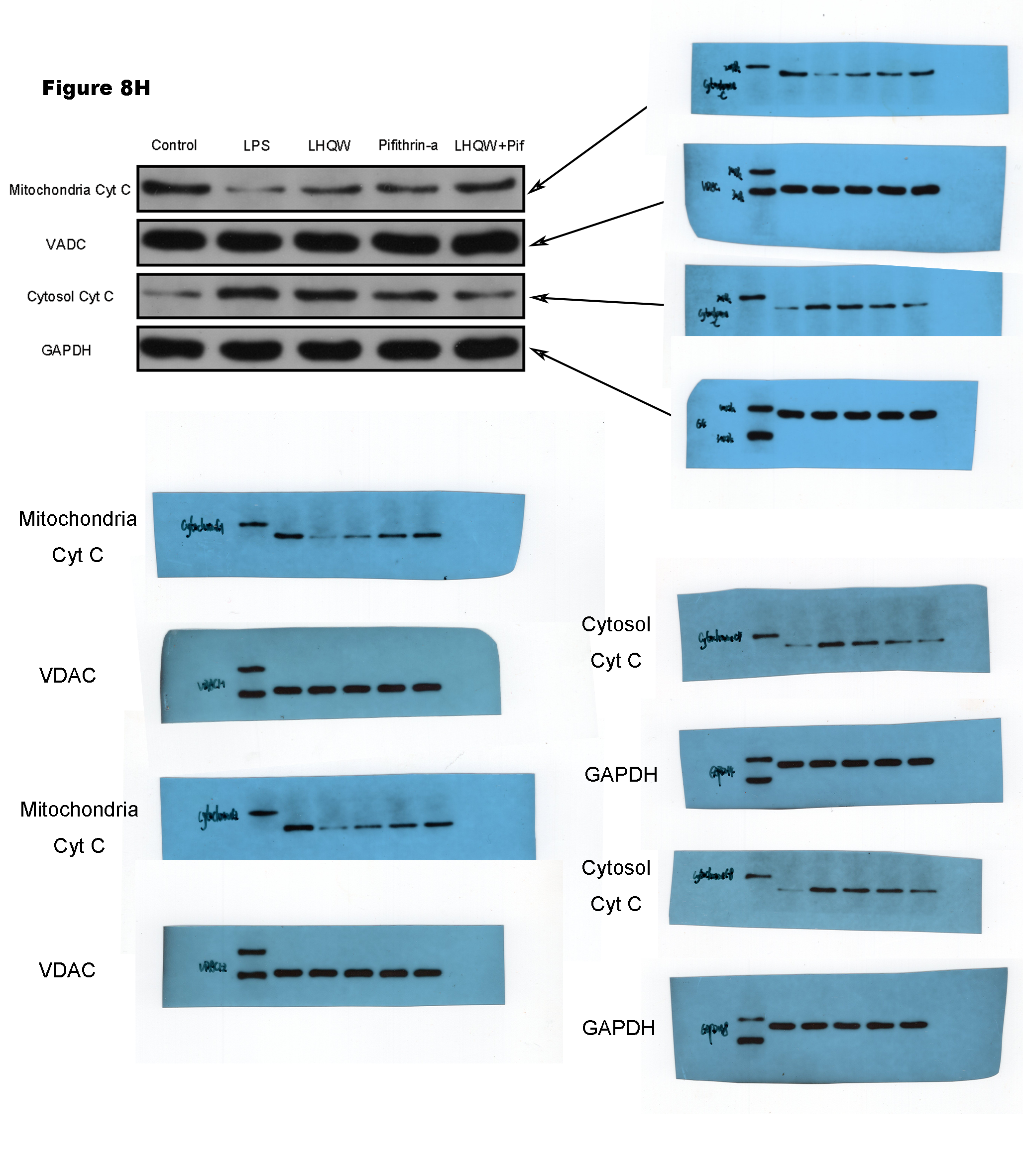

Supplement: Supplementary file 3 [file Table7.DOCX]

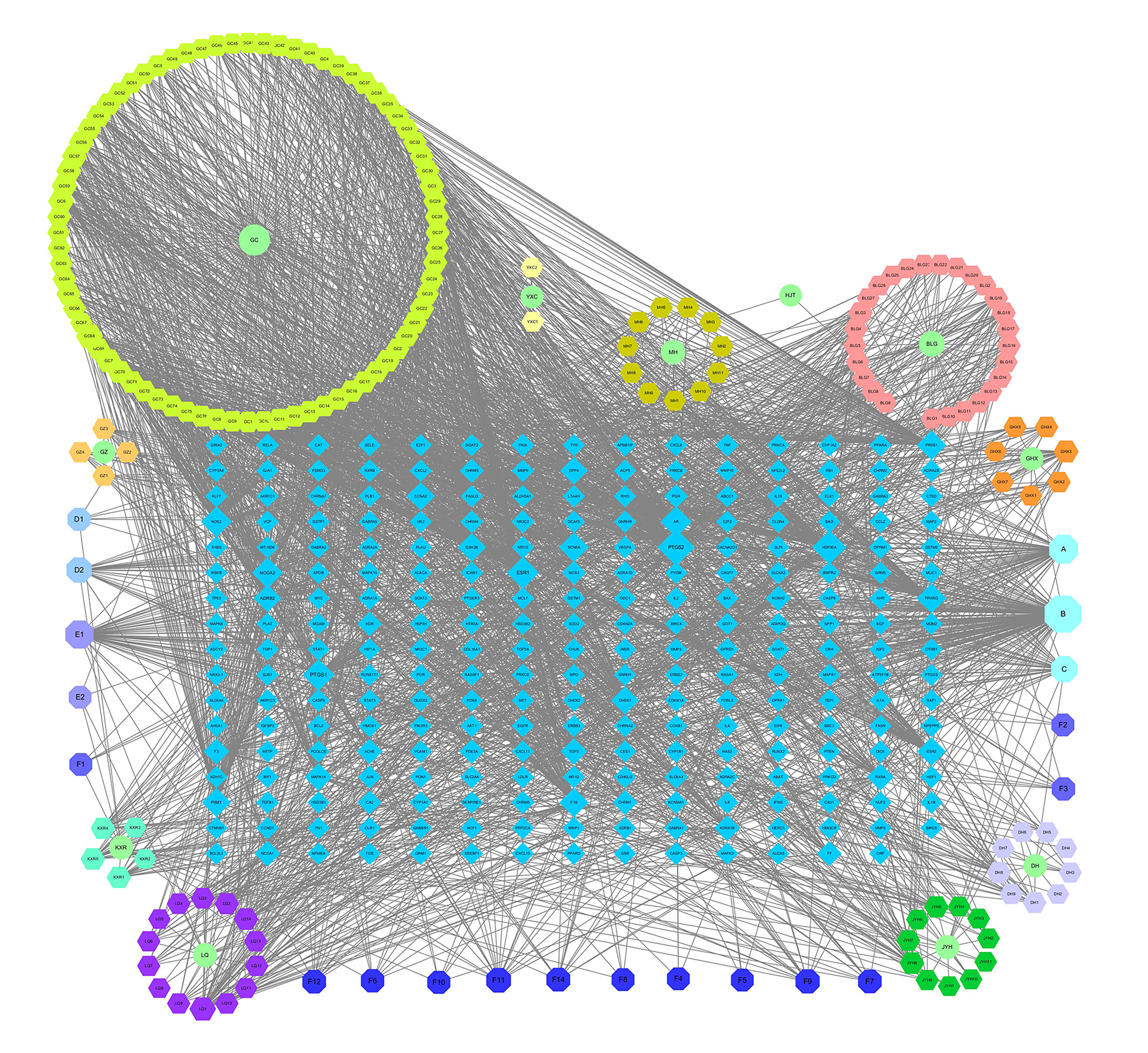

Supplement: Supplementary file 4 [file Image1.TIF]
