# Supplementary material for: Mechanisms Underlying the Effects of Lianhua Qingwen on Sepsis-Induced Acute Lung Injury: A Network Pharmacology Approach
Source: Front Pharmacol. 2021 Oct 14;12:717652. doi: 10.3389/fphar.2021.717652 (PMC8551812; doi:10.3389/fphar.2021.717652)
Supplement: Supplementary file 5 [file Table2.DOCX]

**Target genes of bioactive compounds in LHQW**

| Gene Name | Mol ID |
| --- | --- |
| PTGS2 | MOL001792 MOL000358 MOL000449 MOL000492 MOL002311 MOL004841 MOL004903 MOL004908 MOL005017 MOL000422 MOL004328 MOL000098 MOL005573 MOL001494 MOL000006 MOL001756 MOL001833 MOL001689 MOL001820 MOL001814 MOL001782 MOL001781 MOL001735 MOL001798 MOL001736 MOL001767 MOL001722 MOL001793 MOL001733 MOL001721 MOL001803 MOL001779 MOL001734 MOL001828 MOL001783 MOL001810 MOL010921 MOL007207 MOL012922 MOL002235 MOL002268 MOL002281 MOL000471 MOL000096 MOL001484 MOL000239 MOL002565 MOL000354 MOL003656 MOL003896 MOL000392 MOL000417 MOL004805 MOL004806 MOL004808 MOL004810 MOL004811 MOL004814 MOL004815 MOL004820 MOL004824 MOL004827 MOL004828 MOL004829 MOL004833 MOL004835 MOL004838 MOL004848 MOL004849 MOL004855 MOL004856 MOL004857 MOL004863 MOL004864 MOL004866 MOL004879 MOL004883 MOL004884 MOL004885 MOL004891 MOL004898 MOL004904 MOL004907 MOL004910 MOL004911 MOL004912 MOL004915 MOL004924 MOL004935 MOL004941 MOL004945 MOL004948 MOL004949 MOL004957 MOL004959 MOL004961 MOL004966 MOL000497 MOL004974 MOL004978 MOL004980 MOL004988 MOL004989 MOL004990 MOL004991 MOL004993 MOL000500 MOL005000 MOL005001 MOL005003 MOL005007 MOL005008 MOL005012 MOL005016 MOL005018 MOL005020 MOL001040 MOL002610 MOL002614 MOL005911 MOL005916 MOL005918 MOL005921 MOL002914 MOL003006 MOL003014 MOL002773 MOL003044 MOL003095 MOL003117 MOL000173 MOL003283 MOL003290 MOL003295 MOL003306 MOL003308 MOL003322 MOL003330 MOL003370 MOL000522 MOL000791 MOL010788 MOL002823 MOL010489 MOL004798 MOL002881 MOL004576 MOL005190 MOL005842 MOL007214 |
| HSP90A | MOL001792 MOL000358 MOL000492 MOL004841 MOL005017 MOL000422 MOL004328 MOL000098 MOL005573 MOL000006 MOL001689 MOL001820 MOL001814 MOL001782 MOL001735 MOL001798 MOL001736 MOL001767 MOL001722 MOL001793 MOL001733 MOL001721 MOL001803 MOL001779 MOL001734 MOL001828 MOL001783 MOL007207 MOL012922 MOL002235 MOL002268 MOL002281 MOL000471 MOL000096 MOL001484 MOL000239 MOL002565 MOL000354 MOL003656 MOL003896 MOL000392 MOL000417 MOL004808 MOL004810 MOL004811 MOL004814 MOL004827 MOL004828 MOL004829 MOL004835 MOL004838 MOL004848 MOL004849 MOL004856 MOL004857 MOL004863 MOL004864 MOL004866 MOL004882 MOL004883 MOL004885 MOL004898 MOL004907 MOL004910 MOL004911 MOL004913 MOL004914 MOL004915 MOL004935 MOL004941 MOL004945 MOL004949 MOL004959 MOL004961 MOL004966 MOL000497 MOL004974 MOL004980 MOL004989 MOL004990 MOL004991 MOL004993 MOL000500 MOL005000 MOL005001 MOL005003 MOL005007 MOL005008 MOL005016 MOL005020 MOL001040 MOL005911 MOL005916 MOL005918 MOL005921 MOL002914 MOL003044 MOL003095 MOL003111 MOL000173 MOL003283 MOL003290 MOL003295 MOL003306 MOL003308 MOL003322 MOL003330 MOL003370 MOL000522 MOL000791 MOL010788 MOL002823 MOL010489 MOL004798 MOL002881 MOL004576 MOL005190 MOL007214 |
| ESR1 | MOL001792 MOL000492 MOL002311 MOL004841 MOL004908 MOL005017 MOL004328 MOL001833 MOL001782 MOL001767 MOL001793 MOL001779 MOL001810 MOL002281 MOL000096 MOL002565 MOL000354 MOL003656 MOL003896 MOL000392 MOL000417 MOL004805 MOL004806 MOL004808 MOL004810 MOL004811 MOL004814 MOL004815 MOL004820 MOL004824 MOL004827 MOL004828 MOL004829 MOL004833 MOL004835 MOL004838 MOL004848 MOL004849 MOL004855 MOL004856 MOL004857 MOL004863 MOL004864 MOL004879 MOL004882 MOL004883 MOL004884 MOL004885 MOL004891 MOL004898 MOL004904 MOL004907 MOL004910 MOL004911 MOL004912 MOL004913 MOL004914 MOL004915 MOL004935 MOL004941 MOL004945 MOL004948 MOL004949 MOL004957 MOL004959 MOL004961 MOL004966 MOL000497 MOL004974 MOL004978 MOL004980 MOL004988 MOL004989 MOL004990 MOL004991 MOL004993 MOL000500 MOL005000 MOL005001 MOL005003 MOL005007 MOL005008 MOL005012 MOL005016 MOL005018 MOL005020 MOL001040 MOL002605 MOL005916 MOL005921 MOL003014 MOL003044 MOL003095 MOL003111 MOL000173 MOL003283 MOL003290 MOL010788 |
| PTGS1 | MOL001792 MOL000358 MOL000449 MOL000492 MOL004841 MOL000422 MOL004328 MOL000098 MOL005573 MOL001494 MOL000006 MOL001756 MOL001689 MOL001820 MOL001814 MOL001782 MOL001781 MOL001735 MOL001798 MOL001736 MOL001767 MOL001793 MOL001733 MOL001721 MOL001803 MOL001779 MOL001828 MOL001810 MOL005030 MOL007207 MOL012922 MOL002268 MOL002281 MOL000471 MOL000096 MOL001484 MOL000239 MOL002565 MOL000354 MOL003896 MOL000392 MOL000417 MOL004810 MOL004815 MOL004820 MOL004828 MOL004829 MOL004835 MOL004885 MOL004891 MOL004907 MOL004910 MOL004911 MOL004912 MOL004941 MOL004945 MOL004957 MOL004959 MOL004961 MOL004966 MOL000497 MOL004974 MOL004978 MOL004980 MOL004990 MOL004991 MOL000500 MOL005003 MOL005007 MOL005016 MOL001040 MOL002610 MOL002614 MOL005911 MOL005916 MOL005918 MOL005921 MOL001495 MOL002914 MOL003044 MOL003095 MOL000173 MOL003283 MOL003295 MOL003306 MOL003370 MOL000791 MOL010788 MOL002823 MOL010489 MOL004798 MOL002881 MOL004576 MOL005190 MOL005842 MOL007214 |
| AR | MOL004841 MOL004908 MOL005017 MOL000422 MOL000098 MOL005573 MOL000006 MOL001833 MOL001689 MOL001782 MOL001767 MOL001793 MOL001803 MOL001779 MOL002235 MOL000239 MOL000354 MOL003656 MOL003896 MOL000392 MOL000417 MOL004805 MOL004808 MOL004810 MOL004811 MOL004814 MOL004815 MOL004820 MOL004824 MOL004827 MOL004828 MOL004833 MOL004835 MOL004848 MOL004849 MOL004855 MOL004856 MOL004857 MOL004863 MOL004864 MOL004866 MOL004879 MOL004882 MOL004883 MOL004884 MOL004885 MOL004891 MOL004898 MOL004904 MOL004907 MOL004911 MOL004912 MOL004914 MOL004915 MOL004948 MOL004949 MOL004957 MOL004959 MOL004961 MOL004966 MOL000497 MOL004974 MOL004978 MOL004980 MOL004988 MOL004990 MOL004991 MOL000500 MOL005000 MOL005001 MOL005003 MOL005007 MOL005008 MOL005012 MOL005016 MOL005020 MOL005916 MOL005918 MOL005921 MOL003044 MOL003095 MOL003111 MOL003128 MOL000173 MOL003283 MOL000791 MOL002823 MOL004798 |
| NCOA2 | MOL000953 MOL001771 MOL000359 MOL000358 MOL000449 MOL000492 MOL004355 MOL004908 MOL000422 MOL000098 MOL005573 MOL001494 MOL000006 MOL001756 MOL001689 MOL001735 MOL001733 MOL001803 MOL001783 MOL001804 MOL002211 MOL005030 MOL007207 MOL002235 MOL002268 MOL002297 MOL000471 MOL000096 MOL000239 MOL000354 MOL003656 MOL003896 MOL000417 MOL004808 MOL004811 MOL004815 MOL004820 MOL004848 MOL004849 MOL004855 MOL004856 MOL004857 MOL004863 MOL004864 MOL004879 MOL004883 MOL004898 MOL004911 MOL004949 MOL004959 MOL004961 MOL004966 MOL000497 MOL004974 MOL004978 MOL004980 MOL004985 MOL004988 MOL004991 MOL004996 MOL005000 MOL005001 MOL005003 MOL005007 MOL005016 MOL005018 MOL005020 MOL002614 MOL005916 MOL005921 MOL001495 MOL002914 MOL003036 MOL003044 MOL003095 MOL003111 MOL003283 MOL003290 MOL003295 MOL003306 MOL003308 MOL003322 MOL003330 MOL004798 MOL002881 MOL005190 MOL005842 |
| NOS2 | MOL002311 MOL004841 MOL004908 MOL000422 MOL005573 MOL001689 MOL001782 MOL001735 MOL001767 MOL001793 MOL001733 MOL001803 MOL001779 MOL002235 MOL002281 MOL000239 MOL002565 MOL000354 MOL003656 MOL003896 MOL000392 MOL000417 MOL004805 MOL004806 MOL004808 MOL004810 MOL004811 MOL004814 MOL004815 MOL004820 MOL004824 MOL004827 MOL004828 MOL004833 MOL004835 MOL004838 MOL004848 MOL004849 MOL004855 MOL004856 MOL004857 MOL004863 MOL004864 MOL004879 MOL004883 MOL004884 MOL004885 MOL004891 MOL004904 MOL004907 MOL004910 MOL004911 MOL004912 MOL004915 MOL004945 MOL004948 MOL004949 MOL004957 MOL004959 MOL004961 MOL004966 MOL000497 MOL004974 MOL004978 MOL004989 MOL004990 MOL004991 MOL000500 MOL005000 MOL005003 MOL005007 MOL005008 MOL005012 MOL005016 MOL005018 MOL005020 MOL002610 MOL005916 MOL003044 MOL003095 MOL003117 MOL000173 MOL003370 MOL004798 MOL002881 MOL005842 |
| SCN5A | MOL000358 MOL000449 MOL004908 MOL000098 MOL001820 MOL001814 MOL001798 MOL001749 MOL001733 MOL001803 MOL001779 MOL001828 MOL007207 MOL012922 MOL002235 MOL001484 MOL000239 MOL002565 MOL003656 MOL003896 MOL004806 MOL004810 MOL004811 MOL004815 MOL004820 MOL004827 MOL004828 MOL004829 MOL004833 MOL004835 MOL004856 MOL004866 MOL004885 MOL004891 MOL004910 MOL004911 MOL004912 MOL004915 MOL004945 MOL004957 MOL004959 MOL004961 MOL004966 MOL000497 MOL004974 MOL004978 MOL004980 MOL004989 MOL004991 MOL004993 MOL000500 MOL005003 MOL005007 MOL005012 MOL005016 MOL005020 MOL002605 MOL002610 MOL002879 MOL005911 MOL005916 MOL005918 MOL003095 MOL000173 MOL003283 MOL003290 MOL003295 MOL003306 MOL003308 MOL003322 MOL003330 MOL003370 MOL000522 MOL000791 MOL005842 MOL011319 |
| PPARG | MOL002311 MOL004841 MOL004908 MOL005017 MOL000422 MOL000422 MOL004328 MOL000098 MOL000098 MOL000006 MOL001767 MOL000096 MOL000354 MOL003656 MOL003896 MOL000392 MOL000392 MOL000417 MOL004805 MOL004808 MOL004810 MOL004811 MOL004815 MOL004820 MOL004824 MOL004827 MOL004828 MOL004833 MOL004835 MOL004848 MOL004849 MOL004855 MOL004856 MOL004857 MOL004863 MOL004864 MOL004866 MOL004879 MOL004883 MOL004884 MOL004885 MOL004891 MOL004898 MOL004904 MOL004907 MOL004911 MOL004912 MOL004913 MOL004914 MOL004915 MOL004949 MOL004957 MOL004959 MOL004961 MOL004966 MOL000497 MOL004974 MOL004978 MOL004980 MOL004990 MOL004991 MOL000500 MOL005000 MOL005003 MOL005007 MOL005012 MOL005016 MOL005020 MOL005916 MOL005921 MOL003044 MOL003095 MOL000173 MOL003283 MOL002823 |
| PRSS1 | MOL004908 MOL000422 MOL000098 MOL005573 MOL000006 MOL001689 MOL001735 MOL001733 MOL001803 MOL001828 MOL001783 MOL001810 MOL002235 MOL001484 MOL000239 MOL002565 MOL000354 MOL003656 MOL003896 MOL000392 MOL000417 MOL004808 MOL004810 MOL004811 MOL004820 MOL004824 MOL004827 MOL004828 MOL004833 MOL004849 MOL004855 MOL004856 MOL004857 MOL004863 MOL004864 MOL004866 MOL004879 MOL004883 MOL004884 MOL004885 MOL004891 MOL004904 MOL004907 MOL004911 MOL004912 MOL004915 MOL004949 MOL004957 MOL004959 MOL004961 MOL004966 MOL004974 MOL004978 MOL004980 MOL004991 MOL000500 MOL005000 MOL005001 MOL005003 MOL005007 MOL005008 MOL005012 MOL005016 MOL005020 MOL005916 MOL003044 MOL003095 MOL003111 MOL000173 MOL002823 MOL002881 MOL005842 |
| F10 | MOL004903 MOL000098 MOL001733 MOL001803 MOL001734 MOL001828 MOL001783 MOL012922 MOL002235 MOL003656 MOL004805 MOL004806 MOL004808 MOL004810 MOL004811 MOL004815 MOL004820 MOL004824 MOL004827 MOL004828 MOL004829 MOL004833 MOL004848 MOL004849 MOL004855 MOL004856 MOL004857 MOL004863 MOL004864 MOL004866 MOL004879 MOL004883 MOL004884 MOL004885 MOL004904 MOL004910 MOL004911 MOL004912 MOL004915 MOL004935 MOL004945 MOL004949 MOL004959 MOL004966 MOL000497 MOL004974 MOL004978 MOL004980 MOL004988 MOL004989 MOL004993 MOL005000 MOL005001 MOL005003 MOL005007 MOL005008 MOL005012 MOL005018 MOL005020 MOL003095 MOL003283 MOL003290 MOL003295 MOL003306 MOL003308 MOL003322 MOL003330 MOL000522 MOL000791 |
| PIM1 | MOL002311 MOL004841 MOL004908 MOL005017 MOL001782 MOL001781 MOL001767 MOL001793 MOL002565 MOL000354 MOL003656 MOL003896 MOL000392 MOL000417 MOL004805 MOL004806 MOL004808 MOL004810 MOL004811 MOL004814 MOL004815 MOL004820 MOL004824 MOL004828 MOL004833 MOL004848 MOL004849 MOL004855 MOL004856 MOL004857 MOL004863 MOL004864 MOL004866 MOL004879 MOL004883 MOL004884 MOL004885 MOL004891 MOL004898 MOL004904 MOL004907 MOL004911 MOL004912 MOL004915 MOL004948 MOL004949 MOL004957 MOL004959 MOL004966 MOL000497 MOL004974 MOL004978 MOL004980 MOL004988 MOL004990 MOL000500 MOL005000 MOL005001 MOL005003 MOL005007 MOL005008 MOL005012 MOL005016 MOL005018 MOL005020 MOL005916 MOL003283 |
| ESR2 | MOL004841 MOL004908 MOL005573 MOL001803 MOL001779 MOL002235 MOL002281 MOL000239 MOL002565 MOL000354 MOL003656 MOL003896 MOL000392 MOL000417 MOL004805 MOL004806 MOL004808 MOL004810 MOL004811 MOL004814 MOL004815 MOL004820 MOL004824 MOL004833 MOL004835 MOL004848 MOL004849 MOL004856 MOL004857 MOL004864 MOL004879 MOL004882 MOL004884 MOL004885 MOL004891 MOL004907 MOL004911 MOL004912 MOL004913 MOL004915 MOL004945 MOL004957 MOL004959 MOL004961 MOL004966 MOL000497 MOL004974 MOL004978 MOL004988 MOL004990 MOL000500 MOL005000 MOL005003 MOL005007 MOL005008 MOL005012 MOL005016 MOL005018 MOL005020 MOL002610 MOL005916 MOL003095 MOL003283 MOL003370 |
| GSK3B | MOL002311 MOL004841 MOL004908 MOL005017 MOL001782 MOL001767 MOL001793 MOL000354 MOL003656 MOL003896 MOL000392 MOL000417 MOL004805 MOL004808 MOL004810 MOL004811 MOL004814 MOL004815 MOL004820 MOL004824 MOL004827 MOL004828 MOL004833 MOL004835 MOL004848 MOL004849 MOL004856 MOL004857 MOL004863 MOL004864 MOL004882 MOL004884 MOL004885 MOL004891 MOL004898 MOL004907 MOL004911 MOL004912 MOL004913 MOL004914 MOL004915 MOL004948 MOL004949 MOL004957 MOL004959 MOL004961 MOL004966 MOL000497 MOL004974 MOL004978 MOL004990 MOL004991 MOL000500 MOL005000 MOL005003 MOL005007 MOL005008 MOL005012 MOL005016 MOL005916 MOL003044 MOL003095 MOL000173 MOL003283 |
| CCNA2 | MOL002311 MOL004841 MOL004908 MOL005017 MOL001833 MOL001782 MOL001781 MOL001767 MOL001793 MOL002565 MOL000354 MOL003656 MOL003896 MOL000392 MOL000417 MOL004808 MOL004810 MOL004811 MOL004814 MOL004815 MOL004820 MOL004824 MOL004828 MOL004833 MOL004835 MOL004848 MOL004856 MOL004857 MOL004863 MOL004864 MOL004866 MOL004882 MOL004883 MOL004884 MOL004885 MOL004891 MOL004898 MOL004904 MOL004907 MOL004912 MOL004913 MOL004915 MOL004949 MOL004957 MOL004959 MOL004966 MOL000497 MOL004974 MOL004978 MOL000500 MOL005000 MOL005001 MOL005003 MOL005007 MOL005008 MOL005012 MOL005016 MOL005020 MOL005916 MOL003283 |
| CHEK1 | MOL002311 MOL004841 MOL004908 MOL005017 MOL005573 MOL001689 MOL001782 MOL001793 MOL001803 MOL002281 MOL000239 MOL000354 MOL003656 MOL003896 MOL000392 MOL000417 MOL004811 MOL004814 MOL004815 MOL004820 MOL004824 MOL004827 MOL004828 MOL004833 MOL004835 MOL004849 MOL004855 MOL004856 MOL004857 MOL004863 MOL004864 MOL004866 MOL004879 MOL004883 MOL004884 MOL004907 MOL004912 MOL004913 MOL004914 MOL004915 MOL004957 MOL004966 MOL000497 MOL004974 MOL004978 MOL004990 MOL004991 MOL000500 MOL005000 MOL005012 MOL005016 MOL005020 MOL005916 MOL003044 MOL003095 MOL000173 MOL003283 MOL003370 |
| MAPK14 | MOL002311 MOL004841 MOL004908 MOL005017 MOL001782 MOL001767 MOL001793 MOL000354 MOL003656 MOL003896 MOL000392 MOL000417 MOL004805 MOL004810 MOL004811 MOL004814 MOL004815 MOL004820 MOL004824 MOL004828 MOL004833 MOL004835 MOL004848 MOL004849 MOL004863 MOL004864 MOL004883 MOL004891 MOL004898 MOL004907 MOL004911 MOL004912 MOL004913 MOL004914 MOL004915 MOL004957 MOL004959 MOL004961 MOL004966 MOL000497 MOL004974 MOL004978 MOL004990 MOL004991 MOL000500 MOL005000 MOL005003 MOL005012 MOL005016 MOL005020 MOL005916 MOL003044 MOL003095 MOL000173 MOL003283 |
| DPP4 | MOL000422 MOL000098 MOL005573 MOL000006 MOL001689 MOL001735 MOL001733 MOL001803 MOL001779 MOL002235 MOL000239 MOL002565 MOL000354 MOL003656 MOL003896 MOL000392 MOL000417 MOL004808 MOL004811 MOL004824 MOL004828 MOL004849 MOL004856 MOL004857 MOL004864 MOL004866 MOL004879 MOL004883 MOL004907 MOL004912 MOL004915 MOL004948 MOL004957 MOL004961 MOL004980 MOL004990 MOL004991 MOL000500 MOL005000 MOL005008 MOL005012 MOL005016 MOL005916 MOL005918 MOL003006 MOL003044 MOL003095 MOL003111 MOL003117 MOL000173 MOL003370 MOL002823 MOL002881 MOL005842 |
| ADRB2 | MOL001792 MOL000358 MOL000449 MOL004841 MOL004908 MOL000098 MOL001689 MOL001820 MOL001749 MOL001803 MOL010921 MOL007207 MOL012922 MOL001484 MOL002565 MOL003896 MOL000392 MOL000417 MOL004833 MOL004835 MOL004857 MOL004866 MOL004891 MOL004911 MOL004941 MOL004945 MOL004957 MOL004959 MOL004966 MOL000497 MOL004974 MOL004978 MOL004980 MOL004991 MOL000500 MOL005003 MOL005020 MOL002610 MOL002614 MOL002879 MOL005911 MOL005916 MOL005918 MOL003095 MOL000173 MOL003283 MOL003290 MOL003295 MOL003306 MOL003308 MOL003322 MOL003330 MOL000522 MOL011319 |
| RXRA | MOL001792 MOL000449 MOL000492 MOL004908 MOL000098 MOL005573 MOL001820 MOL001814 MOL001782 MOL001781 MOL001793 MOL001779 MOL010921 MOL007207 MOL012922 MOL001484 MOL002565 MOL003896 MOL000392 MOL000417 MOL004811 MOL004815 MOL004820 MOL004828 MOL004829 MOL004833 MOL004838 MOL004891 MOL004911 MOL004912 MOL004941 MOL004957 MOL004959 MOL004974 MOL004978 MOL004991 MOL000500 MOL005003 MOL005016 MOL002610 MOL005911 MOL005916 MOL005918 MOL000173 MOL003295 MOL003370 MOL004576 |
| TOP2 | MOL000422 MOL000098 MOL001733 MOL001803 MOL001779 MOL001783 MOL001790 MOL002235 MOL002259 MOL002280 MOL002288 MOL003656 MOL004808 MOL004810 MOL004811 MOL004820 MOL004824 MOL004827 MOL004828 MOL004829 MOL004849 MOL004855 MOL004856 MOL004857 MOL004864 MOL004879 MOL004883 MOL004884 MOL004885 MOL004904 MOL004959 MOL004966 MOL004974 MOL005000 MOL005001 MOL005007 MOL005008 MOL005922 MOL005923 MOL003095 MOL003283 MOL003305 MOL000791 |
| ADRA1B | MOL000358 MOL000449 MOL004908 MOL000422 MOL001721 MOL001803 MOL001779 MOL007207 MOL012922 MOL001484 MOL002565 MOL003896 MOL004815 MOL004829 MOL004833 MOL004835 MOL004857 MOL004891 MOL004945 MOL004959 MOL004966 MOL000497 MOL004974 MOL004978 MOL004991 MOL000500 MOL005003 MOL002605 MOL005911 MOL003290 MOL003295 MOL003306 MOL003322 MOL003330 MOL005842 MOL011319 |
| NCOA1 | MOL000449 MOL004908 MOL005573 MOL001689 MOL001735 MOL001733 MOL001803 MOL000354 MOL003896 MOL004820 MOL004829 MOL004833 MOL004835 MOL004849 MOL004885 MOL004891 MOL004959 MOL004966 MOL004974 MOL004978 MOL004993 MOL005007 MOL005916 MOL003044 MOL003095 MOL003111 MOL003295 MOL003306 MOL003308 MOL003322 MOL000522 MOL002881 MOL005842 MOL003851 MOL004350 |
| ACHE | MOL004908 MOL000422 MOL000098 MOL001735 MOL001803 MOL001779 MOL010921 MOL000354 MOL003896 MOL000392 MOL004808 MOL004811 MOL004824 MOL004827 MOL004833 MOL004856 MOL004884 MOL004885 MOL004904 MOL004912 MOL004924 MOL004974 MOL004978 MOL004991 MOL005003 MOL005007 MOL005008 MOL002614 MOL005916 MOL000791 MOL002823 MOL005842 |
| KCNH2 | MOL000358 MOL000098 MOL001803 MOL001828 MOL001783 MOL012922 MOL004805 MOL004806 MOL004811 MOL004849 MOL004855 MOL004864 MOL004879 MOL004891 MOL004959 MOL004966 MOL004974 MOL004978 MOL005003 MOL005007 MOL003095 MOL003290 MOL003295 MOL003306 MOL003308 MOL003322 MOL003330 MOL000522 MOL000791 |
| CHRM1 | MOL000358 MOL000449 MOL004908 MOL000422 MOL001820 MOL001749 MOL001779 MOL010921 MOL007207 MOL012922 MOL001484 MOL002565 MOL003896 MOL000392 MOL004833 MOL004835 MOL004891 MOL004957 MOL000497 MOL004978 MOL000500 MOL005003 MOL002605 MOL005918 MOL003330 MOL011319 |
| F7 | MOL004903 MOL000422 MOL000098 MOL001733 MOL001803 MOL001828 MOL002235 MOL000354 MOL004808 MOL004824 MOL004827 MOL004828 MOL004829 MOL004849 MOL004857 MOL004866 MOL004885 MOL004904 MOL004966 MOL004974 MOL004989 MOL004993 MOL005007 MOL005008 MOL003283 MOL003290 |
| PDE3A | MOL000358 MOL004841 MOL001689 MOL001820 MOL001814 MOL010921 MOL007207 MOL002565 MOL003896 MOL000392 MOL000417 MOL004828 MOL004829 MOL004835 MOL004910 MOL004941 MOL004945 MOL004957 MOL004991 MOL000500 MOL005911 MOL000173 MOL003290 MOL003295 MOL011319 |
| KDR | MOL002311 MOL004903 MOL005017 MOL001810 MOL002235 MOL004808 MOL004824 MOL004828 MOL004848 MOL004849 MOL004855 MOL004857 MOL004879 MOL004883 MOL004904 MOL004935 MOL004959 MOL004966 MOL005001 MOL005007 MOL000173 MOL000522 MOL000791 |
| CHRM3 | MOL000358 MOL000449 MOL001749 MOL001779 MOL010921 MOL007207 MOL012922 MOL001484 MOL002565 MOL003896 MOL004891 MOL004978 MOL005003 MOL002605 MOL002610 MOL002614 MOL002879 MOL005918 MOL003290 MOL003330 |
| NOS3 | MOL000422 MOL000098 MOL001735 MOL001779 MOL000354 MOL003896 MOL000392 MOL004828 MOL004829 MOL004910 MOL004959 MOL004978 MOL004991 MOL005000 MOL005003 MOL002610 MOL002614 MOL003095 MOL003283 |
| PGR | MOL000953 MOL001771 MOL000359 MOL000358 MOL000449 MOL001755 MOL000211 MOL004355 MOL000422 MOL001800 MOL001804 MOL010921 MOL002297 MOL001040 MOL003036 MOL005043 MOL003851 |
| GABRA1 | MOL000358 MOL000449 MOL000422 MOL000098 MOL001820 MOL001814 MOL001782 MOL000354 MOL003896 MOL004941 MOL004991 MOL002610 MOL005918 MOL003006 MOL003117 MOL000173 MOL002823 |
| CA2 | MOL004841 MOL001722 MOL001750 MOL010921 MOL012922 MOL004815 MOL004835 MOL000497 MOL005921 MOL005923 MOL003006 MOL003111 MOL003128 MOL003283 MOL004798 |
| SLC6A3 | MOL000449 MOL010921 MOL007207 MOL012922 MOL002565 MOL003896 MOL000392 MOL004835 MOL004957 MOL000497 MOL004978 MOL000500 MOL003290 MOL011319 |
| SLC6A4 | MOL001792 MOL000358 MOL010921 MOL007207 MOL012922 MOL002565 MOL003896 MOL000392 MOL004835 MOL004941 MOL004957 MOL000500 MOL005911 |
| ADRA1D | MOL010921 MOL007207 MOL012922 MOL001484 MOL002565 MOL003896 MOL004891 MOL004959 MOL004991 MOL002614 MOL003290 |
| OPRM1 | MOL000358 MOL001779 MOL010921 MOL012922 MOL001484 MOL002565 MOL003896 MOL004891 MOL002605 MOL002610 MOL005918 |
| RELA | MOL000422 MOL004328 MOL000098 MOL000006 MOL001689 MOL000354 MOL000497 MOL005916 MOL000173 MOL004576 |
| NR3C2 | MOL000953 MOL000359 MOL000449 MOL001755 MOL004355 MOL001774 MOL001040 MOL003036 MOL003851 MOL004350 |
| JUN | MOL000358 MOL000422 MOL000098 MOL000006 MOL002268 MOL000392 MOL005916 MOL002773 MOL000173 |
| CASP3 | MOL000358 MOL000422 MOL004328 MOL000098 MOL000006 MOL001689 MOL000471 MOL002773 MOL000173 |
| ADRA1A | MOL000358 MOL000449 MOL010921 MOL007207 MOL012922 MOL002565 MOL000392 MOL000500 MOL002614 |
| PKIA | MOL001792 MOL001756 MOL000471 MOL003896 MOL000392 MOL004835 MOL004941 MOL004957 MOL000500 |
| BCL2 | MOL000358 MOL000422 MOL004328 MOL000098 MOL001689 MOL000497 MOL002773 MOL000173 |
| CHRM5 | MOL001779 MOL010921 MOL012922 MOL002565 MOL003896 MOL005003 MOL002605 MOL003330 |
| CHRM2 | MOL000358 MOL000449 MOL000422 MOL010921 MOL012922 MOL002565 MOL002823 |
| CHRM4 | MOL000358 MOL001721 MOL010921 MOL007207 MOL012922 MOL002565 MOL000500 |
| KCNMA1 | MOL001783 MOL004966 MOL004974 MOL004978 MOL005007 MOL003095 MOL003370 |
| OPRD1 | MOL001721 MOL001779 MOL010921 MOL012922 MOL002565 MOL004891 MOL002605 |
| AKT1 | MOL000422 MOL004328 MOL000098 MOL000006 MOL002773 MOL000173 |
| BAX | MOL000358 MOL000422 MOL000098 MOL001689 MOL000471 MOL000173 |
| TNF | MOL000422 MOL000098 MOL000006 MOL000471 MOL005916 MOL000173 |
| CHRNA7 | MOL000358 MOL000449 MOL001820 MOL002565 MOL003896 MOL004891 |
| SLC6A2 | MOL000449 MOL000422 MOL010921 MOL000791 MOL011319 |
| MMP1 | MOL000422 MOL000098 MOL000006 MOL002773 MOL000173 |
| HMOX1 | MOL000422 MOL000098 MOL000006 MOL002773 MOL005190 |
| ICAM1 | MOL000422 MOL000098 MOL000006 MOL003347 MOL004576 |
| CASP9 | MOL000358 MOL000098 MOL000006 MOL002773 MOL000173 |
| CDKN1A | MOL000098 MOL000006 MOL001689 MOL000471 MOL000173 |
| MAPK1 | MOL004328 MOL000098 MOL000006 MOL000497 MOL005916 |
| RXRB | MOL004908 MOL004891 MOL004978 MOL005003 MOL003295 |
| TP53 | MOL000098 MOL000006 MOL001689 MOL000471 MOL000173 |
| XDH | MOL000422 MOL000098 MOL000006 MOL000354 |
| CYP3A4 | MOL000422 MOL000098 MOL002773 MOL003347 |
| CASP8 | MOL000358 MOL000098 MOL001689 MOL002773 |
| CCND1 | MOL000098 MOL000006 MOL000497 MOL000173 |
| EIF6 | MOL000098 MOL000471 MOL000497 MOL000173 |
| FASN | MOL004328 MOL001689 MOL000471 MOL000096 |
| GSTP1 | MOL000422 MOL004328 MOL000098 MOL000006 |
| LTA4H | MOL000449 MOL003896 MOL004835 MOL005911 |
| PPARD | MOL000098 MOL002235 MOL000354 MOL005007 |
| AHSA1 | MOL000422 MOL000098 MOL000173 |
| CYP1A2 | MOL000422 MOL000098 MOL002773 |
| ADRB1 | MOL000449 MOL007207 MOL003896 |
| CCNB1 | MOL000098 MOL000006 MOL000471 |
| CXCL8 | MOL000098 MOL000173 MOL003347 |
| GJA1 | MOL000098 MOL002773 MOL000791 |
| HAS2 | MOL000492 MOL000422 MOL000098 |
| IL1B | MOL000098 MOL000471 MOL005916 |
| IL6 | MOL000098 MOL000006 MOL000173 |
| INSR | MOL000422 MOL000098 MOL000006 |
| MMP2 | MOL000098 MOL000006 MOL002773 |
| MMP9 | MOL000098 MOL000006 MOL005916 |
| MYC | MOL000098 MOL000471 MOL002773 |
| NR1I2 | MOL000422 MOL000098 MOL003347 |
| PRKCA | MOL000358 MOL000098 MOL000471 |
| RB1 | MOL000098 MOL000006 MOL000497 |
| SLC2A4 | MOL000422 MOL000098 MOL000006 |
| SOD2 | MOL004903 MOL004328 MOL000098 |
| VEGFA | MOL000098 MOL000006 MOL002773 |
| GABRA2 | MOL000358 MOL000422 |
| STAT1 | MOL000422 MOL000098 |
| CYP1A1 | MOL000422 MOL000098 |
| SELE | MOL000422 MOL000098 |
| ADRA2A | MOL000449 MOL007207 |
| ADRA2B | MOL007207 MOL012922 |
| ADRA2C | MOL007207 MOL012922 |
| AHR | MOL000422 MOL000098 |
| ALOX5 | MOL000422 MOL000098 |
| APOB | MOL004328 MOL004576 |
| BCL2L1 | MOL000098 MOL000006 |
| BIRC5 | MOL000098 MOL000006 |
| CASP7 | MOL000006 MOL002773 |
| CAT | MOL000492 MOL004328 |
| CAV1 | MOL000098 MOL002773 |
| CCL2 | MOL000098 MOL000173 |
| CD40LG | MOL000098 MOL000006 |
| CYP1B1 | MOL000422 MOL000098 |
| DIO1 | MOL000422 MOL000098 |
| EGFR | MOL000098 MOL000006 |
| ERBB2 | MOL000098 MOL000006 |
| F3 | MOL000098 MOL002773 |
| FOS | MOL000098 MOL000791 |
| GABRA3 | MOL000358 MOL000449 |
| GSTM1 | MOL000422 MOL000098 |
| GSTM2 | MOL000422 MOL000098 |
| HMGCR | MOL004328 MOL005916 |
| HTR3A | MOL001484 MOL004891 |
| IFNG | MOL000098 MOL000006 |
| IL10 | MOL000098 MOL000006 |
| IL2 | MOL000098 MOL000006 |
| IL4 | MOL000006 MOL000392 |
| MCL1 | MOL000006 MOL000173 |
| MTTP | MOL004328 MOL004576 |
| NCF1 | MOL000098 MOL000354 |
| NFE2L2 | MOL000098 MOL005190 |
| NFKBIA | MOL000098 MOL000006 |
| NR1I3 | MOL000422 MOL000098 |
| NR3C1 | MOL001040 MOL003315 |
| PCNA | MOL000006 MOL000471 |
| PLAU | MOL000449 MOL000098 |
| PON1 | MOL000358 MOL000098 |
| PPARA | MOL004328 MOL000098 |
| PRKCD | MOL000471 MOL000173 |
| PSMD3 | MOL000422 MOL000098 |
| PTGER3 | MOL000098 MOL000173 |
| PYGM | MOL000354 MOL002914 |
| TGFB1 | MOL000358 MOL000098 |
| TOP1 | MOL000098 MOL000006 |
| TOP2A | MOL000098 MOL000006 |
| VCAM1 | MOL000422 MOL000098 |
| CHRNA2 | MOL000358 |
| IKBKB | MOL000422 |
| MAPK8 | MOL000422 |
| ABAT | MOL004328 |
| SOAT1 | MOL004328 |
| MMP3 | MOL000098 |
| EGF | MOL000098 |
| ELK1 | MOL000098 |
| RAF1 | MOL000098 |
| HIF1A | MOL000098 |
| RUNX1T1 | MOL000098 |
| HERC5 | MOL000098 |
| ACACA | MOL000098 |
| DUOX2 | MOL000098 |
| THBD | MOL000098 |
| PTEN | MOL000098 |
| IL1A | MOL000098 |
| CXCL11 | MOL000098 |
| CXCL2 | MOL000098 |
| CLDN4 | MOL000098 |
| HSF1 | MOL000098 |
| CXCL10 | MOL000098 |
| CHUK | MOL000098 |
| RASSF1 | MOL000098 |
| E2F1 | MOL000098 |
| E2F2 | MOL000098 |
| CTSD | MOL000098 |
| IGFBP3 | MOL000098 |
| IGF2 | MOL000098 |
| NKX3-1 | MOL000098 |
| RASA1 | MOL000098 |
| MDM2 | MOL000006 |
| ABCC1 | MOL004328 |
| ACP3 | MOL000098 |
| ADCY2 | MOL000006 |
| ADH1C | MOL000449 |
| ADIPOQ | MOL004328 |
| AKR1C1 | MOL004328 |
| AKR1C3 | MOL000422 |
| ALDH3A1 | MOL000791 |
| APBB1IP | MOL000006 |
| ATP5F1B | MOL000392 |
| BAD | MOL004328 |
| BBC3 | MOL000173 |
| BIRC4 | MOL000006 |
| BMPR2 | MOL000791 |
| CACNA2D1 | MOL003095 |
| CDKN2A | MOL000098 |
| CES1 | MOL004328 |
| CHEK2 | MOL000098 |
| COL16A1 | MOL000098 |
| CRH | MOL000791 |
| CRP | MOL000098 |
| CTNNB1 | MOL002773 |
| CTRB1 | MOL000449 |
| DCAF5 | MOL000098 |
| DGAT2 | MOL004576 |
| ERBB3 | MOL000098 |
| FASLG | MOL001689 |
| FN1 | MOL000173 |
| FOSL2 | MOL000497 |
| GABBR1 | MOL000791 |
| GABRA5 | MOL000358 |
| GNRHR | MOL000791 |
| GOT1 | MOL004328 |
| GRIA2 | MOL000354 |
| GRM5 | MOL000791 |
| GSR | MOL004328 |
| HK2 | MOL000098 |
| HSD3B2 | MOL000392 |
| HSPB1 | MOL000098 |
| IRF1 | MOL000098 |
| KLF7 | MOL000096 |
| LDLR | MOL004328 |
| MAP2 | MOL000358 |
| MAPK10 | MOL002565 |
| MAPK3 | MOL004328 |
| MET | MOL000006 |
| MGAM | MOL000098 |
| MMP10 | MOL002773 |
| MPO | MOL000098 |
| MT-ND6 | MOL000392 |
| MUC1 | MOL000522 |
| NPEPPS | MOL000098 |
| NUF2 | MOL000006 |
| ODC1 | MOL000098 |
| OLR1 | MOL000354 |
| OPRK1 | MOL010921 |
| PCOLCE | MOL000098 |
| PIK3R3 | MOL000173 |
| PLAT | MOL000098 |
| PLB1 | MOL004328 |
| POR | MOL000098 |
| PPP3CA | MOL000422 |
| PRKCB | MOL000098 |
| PRKCE | MOL000471 |
| PTGES | MOL000006 |
| RHO | MOL001735 |
| RUNX2 | MOL000098 |
| SERPINE1 | MOL000098 |
| SLPI | MOL000422 |
| SOAT2 | MOL004328 |
| SPP1 | MOL000098 |
| SREBF1 | MOL004328 |
| STAT3 | MOL000497 |
| TEP1 | MOL000173 |
| TYR | MOL000006 |
| HSD3B1 | MOL000392 |
| GNRH1 | MOL000791 |
| GJB1 | MOL000791 |
| GRM1 | MOL000791 |
| VCP | MOL000791 |
