# Supplementary material for: Mechanisms Underlying the Effects of Lianhua Qingwen on Sepsis-Induced Acute Lung Injury: A Network Pharmacology Approach
Source: Front Pharmacol. 2021 Oct 14;12:717652. doi: 10.3389/fphar.2021.717652 (PMC8551812; doi:10.3389/fphar.2021.717652)
Supplement: Supplementary file 6 [file Table5.DOCX]

Cluster of Compound-ALI overlapping genes

| Cluster | Score | Nodes | Edges | Genes |
| --- | --- | --- | --- | --- |
| 1 | 15.238 | 22 | 160 | MYC, EGF, EGFR, I​​L1A, IL2, IL1B, TNF, CCND1, MAPK3, IL10, CCL2, ESR1, PTEN, TP53, IL6, IL4, STAT3, HMOX1, JUN, PTGS2, CASP3, ERBB2 |
| 2 | 10.526 | 20 | 100 | MAPK1, RELA, SERPINE1, CTNNB1, BCL2L1, CXCL10, PPARG, VEGFA, MMP2, NOS3, AKT1, MAPK8, HIF1A, CXCL8, MMP1, MMP9, TGFB1, IFNG, ICAM1, STAT1 |
| 3 | 4 | 4 | 6 | CDKN1A, RB1, CDKN2A, MDM2 |
