# Supplementary material for: Mechanisms Underlying the Effects of Lianhua Qingwen on Sepsis-Induced Acute Lung Injury: A Network Pharmacology Approach
Source: Front Pharmacol. 2021 Oct 14;12:717652. doi: 10.3389/fphar.2021.717652 (PMC8551812; doi:10.3389/fphar.2021.717652)
Supplement: Supplementary file 7 [file Table3.DOCX]

**Overlapped genes with combined score≥0.7**

| No. | Symbol name | Gene name | degree. |
| --- | --- | --- | --- |
| 1 | AKT1 | RAC-alpha serine/threonine-protein kinase | 52 |
| 2 | TP53 | Cellular tumor antigen p53 | 49 |
| 3 | IL6 | Interleukin-6 | 49 |
| 4 | VEGFA | Vascular endothelial growth factor A | 48 |
| 5 | TNF | Tumor necrosis factor | 48 |
| 6 | JUN | Transcription factor AP-1 | 46 |
| 7 | STAT3 | Signal transducer and activator of transcription 3 | 46 |
| 8 | MAPK8 | Mitogen-activated protein kinase 8 | 43 |
| 9 | MAPK1 | Mitogen-activated protein kinase 1 | 42 |
| 10 | EGF | Pro-epidermal growth factor | 40 |
| 11 | EGFR | Epidermal growth factor receptor | 38 |
| 12 | MMP9 | Matrix metalloproteinase-9 | 37 |
| 13 | IL1B | Interleukin-1 beta | 36 |
| 14 | MAPK3 | Mitogen-activated protein kinase 3 | 36 |
| 15 | MYC | Myc proto-oncogene protein | 33 |
| 16 | CXCL8 | Interleukin-8 | 33 |
| 17 | CCL2 | C-C motif chemokine 2 | 33 |
| 18 | PTGS2 | Prostaglandin G/H synthase 2 | 33 |
| 19 | ICAM1 | Intercellular adhesion molecule 1 | 32 |
| 20 | CCND1 | G1/S-specific cyclin-D1 | 31 |
| 21 | CASP3 | Caspase-3 | 30 |
| 22 | IL10 | Interleukin-10 | 29 |
| 23 | RELA | Transcription factor p65 | 29 |
| 24 | PTEN | Phosphatase and tensin homolog | 28 |
| 25 | ESR1 | Estrogen receptor | 28 |
| 26 | STAT1 | Signal transducer and activator of transcription 1-alpha/beta | 28 |
| 27 | FN1 | Fibronectin type III domain containing | 28 |
| 28 | MMP2 | 72 kDa type IV collagenase | 27 |
| 29 | NOS3 | Nitric oxide synthase, endothelial | 25 |
| 30 | CTNNB1 | Catenin beta-1 | 25 |
| 31 | ERBB2 | Receptor tyrosine-protein kinase erbB-2 | 25 |
| 32 | IL2 | Interleukin-2 | 25 |
| 33 | IL4 | Interleukin-4; | 24 |
| 34 | HMOX1 | Heme oxygenase 1 | 23 |
| 35 | TGFB1 | Transforming growth factor beta-1 | 23 |
| 36 | BCL2L1 | Bcl-2-like protein 1 | 22 |
| 37 | IFNG | Interferon gamma | 22 |
| 38 | PPARG | Peroxisome proliferator-activated receptor gamma | 22 |
| 39 | SERPINE1 | Plasminogen activator inhibitor 1 | 21 |
| 40 | MMP1 | Interstitial collagenase | 20 |
| 41 | HIF1A | Hypoxia-inducible factor 1-alpha | 18 |
| 42 | CAV1 | Caveolin-1 | 18 |
| 43 | CRP | C-reactive protein | 18 |
| 44 | CDKN2A | Cyclin-dependent kinase inhibitor 2A | 16 |
| 45 | IGF2 | Insulin-like growth factor II | 16 |
| 46 | MDM2 | E3 ubiquitin-protein ligase Mdm2 | 15 |
| 47 | IL1A | Interleukin-1 alpha | 15 |
| 48 | CXCL10 | C-X-C motif chemokine 10 | 15 |
| 49 | NOS2 | Nitric oxide synthase, inducible | 15 |
| 50 | CDKN1A | Cyclin-dependent kinase inhibitor 1 | 14 |
| 51 | CASP8 | Caspase-8 | 14 |
| 52 | RB1 | Retinoblastoma-associated protein | 14 |
| 53 | KDR | Vascular endothelial growth factor receptor 2 | 12 |
| 54 | BCL2 | Apoptosis regulator Bcl-2 | 12 |
| 55 | IRF1 | Interferon regulatory factor 1 | 12 |
| 56 | CD40LG | CD40 ligand | 12 |
| 57 | CAT | Catalase | 12 |
| 58 | FASLG | Tumor necrosis factor ligand superfamily member 6 | 11 |
| 59 | MPO | Myeloperoxidase | 11 |
| 60 | ERBB3 | Receptor tyrosine-protein kinase erbB-3 | 10 |
| 61 | SPP1 | Osteopontin | 10 |
| 62 | NFE2L2 | Nuclear factor erythroid 2-related factor 2 | 9 |
| 63 | RAF1 | RAF proto-oncogene serine/threonine-protein kinase | 9 |
| 64 | ESR2 | Estrogen receptor beta | 9 |
| 65 | F3 | Tissue factor | 9 |
| 66 | MUC1 | Mucin-1 | 8 |
| 67 | PLAT | Tissue-type plasminogen activator | 7 |
| 68 | BAX | Apoptosis regulator BAX | 7 |
| 69 | CHEK2 | Serine/threonine-protein kinase Chk2 | 7 |
| 70 | PLAU | Urokinase-type plasminogen activator | 6 |
| 71 | GJA1 | Gap junction alpha-1 protein | 6 |
| 72 | GSTP1 | Glutathione S-transferase P | 5 |
| 73 | THBD | Thrombomodulin | 5 |
| 74 | ADRB2 | Beta-2 adrenergic receptor | 4 |
| 75 | CYP1A1 | Cytochrome P450 1A1 | 3 |
| 76 | ALOX5 | Arachidonate 5-lipoxygenase | 2 |
| 77 | GSTM1 | Glutathione S-transferase Mu 1 | 2 |
| 78 | BMPR2 | Bone morphogenetic protein receptor type-2 | 1 |
