# Supplementary material for: Mechanisms Underlying the Effects of Lianhua Qingwen on Sepsis-Induced Acute Lung Injury: A Network Pharmacology Approach
Source: Front Pharmacol. 2021 Oct 14;12:717652. doi: 10.3389/fphar.2021.717652 (PMC8551812; doi:10.3389/fphar.2021.717652)
Supplement: Supplementary file 8 [file Table4.DOCX]

Top 20 of KEGG pathway analysis based on 80 overlapping genes

| No. | Pathway ID | Pathway Name | P value | Count | Gene Name |
| --- | --- | --- | --- | --- | --- |
| 1 | hsa05200 | Pathways in cancer | 2.14E-62 | 49 | AKT1,FASLG,BAX,CCND1,BCL2,BCL2L1,  CASP3,CASP8,CDKN1A,CDKN2A,CTNNB1,  EGF,EGFR,ERBB2,ESR1,ESR2,FN1,GSTM1,  GSTP1,HIF1A,HMOX1,IFNG,IGF2,IL2,IL4,IL6,  CXCL8,JUN,MDM2,MMP1,MMP2,MMP9,MYC,  NFE2L2,NOS2,PPARG,MAPK1,MAPK3,  MAPK8,PTEN,PTGS2,RAF1,RB1,RELA,STAT1,  STAT3,TGFB1,TP53,VEGFA |
| 2 | ko04933 | AGE-RAGE signaling pathway in diabetic complications | 1.78E-47 | 27 | AKT1,BAX,CCND1,BCL2,CASP3,F3,FN1,  ICAM1,IL1A,IL1B,IL6,CXCL8,JUN,MMP2,NOS3,SERPINE1,MAPK1,MAPK3,MAPK8,RELA,  CCL2,STAT1,STAT3,TGFB1,THBD,TNF,VEGFA |
| 3 | hsa05205 | Proteoglycans in cancer | 3.80E-41 | 29 | AKT1,FASLG,CCND1,CASP3,CAV1,CDKN1A,  CTNNB1,EGFR,ERBB2,ERBB3,ESR1,FN1,  HIF1A,IGF2,IL6,KDR,MDM2,MMP2,MMP9,  MYC,PLAU,MAPK1,MAPK3,RAF1,STAT3,  TGFB1,TNF,TP53,VEGFA |
| 4 | ko05418 | Fluid shear stress and atherosclerosis | 1.17E-40 | 26 | AKT1,BCL2,BMPR2,CAV1,CTNNB1,GSTM1,  GSTP1,HMOX1,ICAM1,IFNG,IL1A,IL1B,JUN,  KDR,MMP2,MMP9,NFE2L2,NOS3,PLAT,  MAPK8,RELA,CCL2,THBD,TNF,TP53,VEGFA |
| 5 | hsa05160 | Hepatitis C | 1.45E-36 | 25 | AKT1,FASLG,BAX,CCND1,CASP3,CASP8,  CDKN1A,CTNNB1,EGF,EGFR,IFNG,CXCL8,  CXCL10,IRF1,MYC,MAPK1,MAPK3,MAPK8,  RAF1,RB1,RELA,STAT1,STAT3,TNF,TP53 |
| 6 | hsa04151 | PI3K-Akt signaling pathway | 1.20E-30 | 27 | AKT1,FASLG,CCND1,BCL2,BCL2L1,CDKN1A,  EGF,EGFR,ERBB2,ERBB3,FN1,IGF2,IL2,IL4,IL6,KDR,MDM2,MYC,NOS3,MAPK1,MAPK3,PTEN,RAF1,RELA,SPP1,TP53,VEGFA |
| 7 | hsa05210 | Colorectal cancer | 1.62E-26 | 17 | AKT1,BAX,CCND1,BCL2,CASP3,CDKN1A,  CTNNB1,EGF,EGFR,JUN,MYC,MAPK1,MAPK3,MAPK8,RAF1,TGFB1,TP53 |
| 8 | hsa05169 | Epstein-Barr virus infection | 3.02E-25 | 22 | AKT1,BAX,CCND1,BCL2,CASP3,CASP8,  CDKN1A,ICAM1,IFNG,IL6,IL10,CXCL10,JUN,  MDM2,MYC,MAPK8,RB1,RELA,STAT1,STAT3,TNF,TP53 |
| 9 | hsa05222 | Small cell lung cancer | 3.09E-22 | 15 | AKT1,BAX,CCND1,BCL2,BCL2L1,CASP3,  CDKN1A,FN1,MYC,NOS2,PTEN,PTGS2,RB1,  RELA,TP53 |
| 10 | ko04060 | Cytokine-cytokine receptor interaction | 1.82E-21 | 19 | FASLG,BMPR2,CD40LG,EGF,EGFR,  IFNG,IL1A,IL1B,IL2,IL4,IL6,CXCL8,IL10,  CXCL10,KDR,CCL2,TGFB1,TNF,VEGFA |
| 11 | hsa05202 | Transcriptional misregulation in cancer | 7.59E-16 | 14 | BAX,BCL2L1,CDKN1A,IL6,CXCL8,MDM2,  MMP9,MPO,MYC,PLAT,PLAU,PPARG,RELA,  TP53 |
| 12 | ko04064 | NF-kappa B signaling pathway | 1.70E-13 | 10 | BCL2,BCL2L1,CD40LG,ICAM1,  IL1B,CXCL8,PLAU,PTGS2,RELA,TNF |
| 13 | ko05330 | Allograft rejection | 1.41E-11 | 7 | FASLG,CD40LG,IFNG,IL2,IL4,IL10,TNF |
| 14 | ko05014 | Amyotrophic lateral sclerosis (ALS) | 1.26E-10 | 7 | BAX,BCL2,BCL2L1,CASP3,CAT,TNF,TP53 |
| 15 | hsa05020 | Prion disease | 6.76E-10 | 6 | BAX,IL1A,IL1B,IL6,MAPK1,MAPK3 |
| 16 | hsa04931 | insulin resistance | 1.26E-09 | 8 | AKT1,IL6,NOS3,MAPK8,PTEN,RELA,STAT3,  TNF |
| 17 | ko04350 | TGF-beta signaling pathway | 4.47E-09 | 7 | BMPR2,IFNG,MYC,MAPK1,MAPK3,TGFB1,TNF |
| 18 | ko04137 | Mitophagy - animal | 3.24E-08 | 6 | BCL2L1,HIF1A,JUN,MAPK8,RELA,TP53 |
| 19 | hsa05416 | Viral myocarditis | 5.13E-08 | 6 | CCND1,CASP3,CASP8,CAV1,CD40LG,ICAM1 |
| 20 | hsa04610 | Complement and coagulation cascades | 2.19E-07 | 6 | F3,CXCL10,SERPINE1,PLAT,PLAU,THBD |
