# Supplementary material for: Mechanisms Underlying the Effects of Lianhua Qingwen on Sepsis-Induced Acute Lung Injury: A Network Pharmacology Approach
Source: Front Pharmacol. 2021 Oct 14;12:717652. doi: 10.3389/fphar.2021.717652 (PMC8551812; doi:10.3389/fphar.2021.717652)
Supplement: Supplementary file 9 [file Table6.DOCX]

**Source and batch number**

Lianhua Qingwen were produced by Shijiazhuang Yiling Pharmaceutical Co., Ltd, China (B2001019).

**Contents and proportions**

Lianhua Qingwen consists of 11 herbs and two mineral Chinese medicines, including *Forsythia suspensa （Thunb.）Vahl* (Lianqiao), *Lonicera japonica Thunb.* (Jinyinhua), *Ephedra sinica Stapf* (Mahuang), *Isatis tinctoria L.* (Banlangen), *Pogostemon cablin（Blanco）Benth.* (Guanghuoxiang), *Rheum palmatum L.* (Dahuang), *Glycyrrhiza uralensis Fisch.* (Gancao), *Dryopteris crassirhizoma Nakai* (Mianmaguanzhong), *Rhodiola crenulata (Hook.f. & Thomson) H.Ohba* (Hongjingtian), *Houttuynia cordata Thunb.* (Yuxingcao), *Prunus sibirica L.* (Kuxingren), *Gypsum* and *l-Menthol*. (Table 1)

| Table 1 Formulation of Lianhuaqingwen capsule（Granule） | | |
| --- | --- | --- |
| Ingredient | Components | % |
| *Forsythia suspensa* （Thunb.）Vahl | Dried Fruit | 12.7 |
| *Lonicera japonica* Thunb. | Dried flower bud or opening flower | 12.7 |
| *Ephedra sinica* Stapf | Dried herbaceous stem | 4.2 |
| *Isatis indigotica* Fort. | Dried root | 12.7 |
| *Pogostemon cablin*（Blanco）Benth. | Dried aerial part | 4.2 |
| *Rheum palmatum* L. | Dried root and rhizome | 2.5 |
| *Glycyrrhiza uralensis* Fisch. | Dried root and rhizome | 4.2 |
| *Dryopteris crassirhizoma* Nakai | Dried rhizome and frond bases | 12.7 |
| *Rhodiola crenulata*（Hook. f. et Thoms. ）H. Ohba | Dried root and rhizome | 4.2 |
| *Houttuynia cordata* Thunb. | Dried aerial part | 12.7 |
| *Prunus sibirica* L. | Dried ripe seed | 4.2 |
| Gypsum | CaSO_4_·2H_2_O | 12.7 |
| l-Menthol | C_10_H_20_O | 0.4 |

**Method of extraction**

Water was added to *Pogostemon cablin（Blanco）Benth.* (Guanghuoxiang) to distill it and collecting the volatile oil, water extract of *Pogostemon cablin（Blanco）Benth.* (Guanghuoxiang) were reserved; *Forsythia suspensa （Thunb.）Vahl* (Lianqiao), *Ephedra sinica Stapf* (Mahuang), *Houttuynia cordata Thunb.* (Yuxingcao) and *Rheum palmatum L.* (Dahuang) were extracted twice with 70% ethanol for 2 hours and 1.5 hours respectively, the extracts were filtered and mixed it together, ethanol is recovered for use; *Lonicera japonica Thunb.* (Jinyinhua), *Gypsum,* *Isatis tinctoria L.* (Banlangen), *Dryopteris crassirhizoma Nakai* (Mianmaguanzhong), *Glycyrrhiza uralensis Fisch.* (Gancao) and *Rhodiola crenulata (Hook.f. & Thomson) H.Ohba* (Hongjingtian) were decocted with water until boiling, then adding *Prunus sibirica L.* (Kuxingren) to be decocted twice for 1.5 hours and 1 hour respectively. The decoction was filtered and mixed together. Then it was added water extract of *Pogostemon cablin（Blanco）Benth.* (Guanghuoxiang) and then were concentrated to a relative density of 1.10～1.15 (60℃), subsequently, ethanol was added to make the alcohol content reach 70 %. Then refrigerating it at 4°C for 24 hours and filtering it and collecting the filtrate. Ethanol were recovered from the filtrate. The filtrate were mixed with the alcohol extracts of above four herbs containing *Forsythia suspensa （Thunb.）Vahl* (Lianqiao). Then the mixture were concentrated to a relative density of 1.15 to 1.20 (60°C), then making it dry by spray dry technology and mixing it with starch to obtain granules. The granules were dried and were sieved out appropriate amount of fine powder. The *l-Menthol* and the volatile oil of *Pogostemon cablin（Blanco）Benth.* (Guanghuoxiang) were dissolved in ethanol, then spraying it into the fine powder, then mixing the fine powder with the above granules and then making it airtight for 30 minutes. Finally, put it into 1000 capsules.

**Quality control**

The fingerprint of Lianhuaqingwen was established by an ultra-performance liquid chromatography (UPLC) method. The UPLC system was a Waters ACQUITY instrument, and the separation was carried out on a Waters HSS T3 column (1.8 μ，100×2.1 mm I.D.). For UPLC analysis, a 2-μl test sample was injected into the column and eluted at 30℃ with a constant flow rate of 0.3 ml/min. In gradient elution the composition of the mobile phases are (solvent A): water with 0.1% phosphoric acid, (solvent B): acetonitrile and (solvent C)：methanol. Chemical standards and Lianhuaqingwen capsule powder were dissolved in 50% (w/w) methanol. All solutions were filtered through Milles 0.22 μm nylon membrane syringe filters before use.

32 common peaks as well as their retention times and peak area rations on UPLC fingerprint can be used as the important parameters of the quality control for Lianhuaqingwen capsule. 9 of the 32 common peaks were identified by compared with chemical standards, they are Neochlorogenic acid (peak 4), chlorogenic acid (peak 6), cryptochlorogenic acid (peak 8), isoforsythoside A (peak 15), forsythoside A (peak 20), quercitrin (peak 23), isochlorogenic acid C (peak 24), forsythin (peak 26), glycyrrhizic acid (peak 31). Representative chromatograms of standards and tested drug and chemical structures of marker compounds are shoun in Fig 1.

**A**


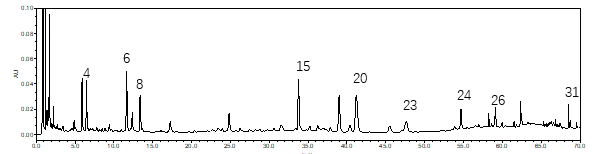


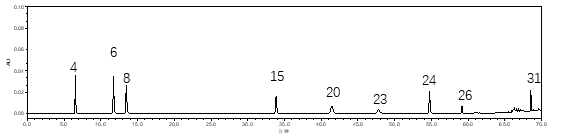


**B**

Fig1. Ultra-performance liquid chromatography (UPLC) analysis of Lianhuaqingwen. (A) Fingerprint of Lianhuaqingwen (upper panel) and analysis of chemical standards (lower panel). (B) Chemical structures of identified compounds in Lianhuaqingwen, corresponding to the common peak numbers.
